# Glutamatergic pathways from medial prefrontal cortex to paraventricular nucleus of thalamus contribute to the methamphetamine-induced conditioned place preference without affecting wakefulness

**DOI:** 10.7150/thno.100688

**Published:** 2025-01-02

**Authors:** Xiang Li, Xing Xu, Quying Feng, Ning Zhou, Yuhong He, Ying Liu, Haoqing Tai, Hee Young Kim, Yu Fan, Xiaowei Guan

**Affiliations:** 1 Department of Human Anatomy and Histoembryology, Nanjing University of Chinese Medicine, Nanjing 210023, China.; 2 Department of Physiology, Yonsei University College of Medicine, Seoul, South Korea.

**Keywords:** METH, PVT, mPFC-PVT glutamatergic pathway, wakefulness, GluN2A

## Abstract

Methamphetamine (METH) is a commonly abused psychostimulant with a high addictive nature. The paraventricular nucleus of thalamus (PVT), a key nucleus for arousal, has attracted much attention in the reward process of substance use. However, at which stage dose the PVT encode the reward process? How to reduce the side-effects of modulating PVT on wakefulness during the treatment of substance use? These issues remain unclear. The goal of the current study is to explore the role of the PVT and the glutamatergic projections from medial prefrontal cortex (mPFC) to PVT in the reward process of METH.

**Methods:** Here, the conditioned place preference (CPP) was used to assess the reward process of METH in male mice, combined with methods of c-Fos mapping, virus-based neural tracing, patch-clamp recording, EEG-EMG recordings, optogenetics and designer receptor exclusively activated by designer drugs (*DREADDs*).

**Results:** The glutamatergic neurons in PVT (PVT^Glu^) were triggered during METH CPP-Test, rather than by METH CPP-Training. Suppressing either PVT^Glu^ or glutamatergic projection from mPFC to PVT efficiently disrupted the acquisition of METH CPP in male mice, mainly mediated by the GluN2A subunit of NMDA receptor. Further, inhibition of PVT^Glu^ affected the rhythm of EEG-EMG, whereas inhibition of glutamatergic projection from mPFC to PVT did not.

**Conclusion:** PVT^Glu^ is involved in the reward process of METH at the retrieval stage of METH-conditioned context, rather than at the stage of encoding association between METH and context. The glutamatergic projections from mPFC to PVT, especially the GluN2A molecule, may be a promising therapeutic target for reducing METH reward, as there are no significant side effects on wakefulness.

## Introduction

Methamphetamine (METH) is a commonly abused psychostimulant with a high addictive nature. Repeated METH intake triggers reward systems in the brain, which contribute to the development of addiction. Recently, the paraventricular nucleus of thalamus (PVT), an integrated nucleus for wakefulness 1, food intake 2, reward 3 and fear memory 4, has been documented as an essential thalamic hub for addiction, such as cocaine sensitization 5, opiate withdrawal 6 and drug seeking 7, 8. It is worth noting that the regulation of PVT carries the risk of interfering with biological rhythms, such as sleep and arousal 9-11. This raises some questions that need to be explored, such as: at which stage does the PVT encode the reward process? How can we reduce the side effects of modulating PVT on wakefulness during treatment of substance use? These questions need to be further explored. As such, it is necessary to explore precise PVT-related pathways that can improve drug rewards without affecting biorhythms.

Previous studies including ours demonstrate the important regulatory role of the medial prefrontal cortex (mPFC) in the process of METH reward 12-17. The PVT is one of the key nuclei innervated by the mPFC, forming mPFC-PVT pathway 18. Inhibition of mPFC-PVT pathway has been reported to disrupt morphine withdrawal memory 19 and cocaine-seeking behavior 20. Otis *et al.* 21 have reported that the mPFC neurons projecting to the PVT play important roles in the acquisition of cue discrimination during the reward-seeking behavior. Taken together, these studies demonstrate that mPFC-PVT pathway may be a promising therapeutic target for the METH reward.

In the current study, to explore the role of PVT and glutamatergic projections from mPFC to PVT in the process of METH reward, METH conditioned place preference (CPP) models were built up in male mice, with a particular focus on their effects on wakefulness.

## Methods and Materials

### Animals

Male C57BL/6 wild type (WT) mice (22~25 g, 8-10 weeks of age) were used. All animals were housed at constant humidity (40-60%) and temperature (24 ± 2 ºC) with a 12 h light/dark cycle (lights on at 7 a.m.) and allowed free access to food and water. All male mice were handled for three days before onset of experiments.

All procedures were carried out in accordance with the National Institutes of Health Guide for the Care and Use of Laboratory Animals and approved by the Institutional Animal Care and Use Committee (IACUC) at Nanjing University of Chinese Medicine, China.

### Drug administration

The METH powder (20 mg/unit, 99.99%) was obtained from China Institute for Food and Drug Control. The stock solution of METH was prepared at 1 mg/mL with sterile saline (0.9% Nacl). The working solution of METH was prepared by diluting the stock solution with sterile saline and the desired concentration was adjusted to 0.1 mg/mL. The solution was prepared fresh before each experiment to ensure stability and consistency of METH.

### Stereotaxic surgery

The male mice were anesthetized with 2% isoflurane and placed on a stereotaxic apparatus (RWD, China). The eyes were covered with eye ointment (Cisen, China) to prevent drying. After shaving the hair and cleaning the incision site with iodine and 70% medical alcohol, the scalp was incised to expose the skull. We dropped 30% hydrogen peroxide onto the skull to corrode connective tissue, and the connective tissue was gently removed from the skull surface with cotton swabs. Small craniotomy holes (~1 mm diameter) were drilled with the help of a microscope (RWD, China) for related virus injection, optic fiber or cannula embedding. The stereotaxic coordinates used in the current study were as following, mPFC (AP + 1.94 mm, ML +/- 0.50 mm, DV - 2.25 mm) and PVT (AP - 1.46 mm, ML 0 mm, DV - 2.95 mm) 1, 22-24. The accuracy of stereotaxis was verified by histological methods. The mice with inaccurate location of viral injection, or fiber optic implantation, or cannula implantation were excluded from the subsequent experiments in this study.

### Tracing virus injection

All viruses in the present study were packaged by BrainVTA (China). The male mice were fixed in a stereotactic frame (RWD, China) under 2% isoflurane anesthesia. A heating pad was used to maintain the body temperature of the mice at 37 °C. The injections were given over 5 min by an infusion pump (Drummond, USA) and left in place for 5 min.

For anterograde tracing 25, a mixture of *rAAV2/9-CaMKIIα-Cre-WPRE-hGH-pA* (100 nL, PT-0220, 5.22E+12 vg/mL, BrainVTA, China) and *rAAV2/9-hSyn-DIO-mGFP-T2A-Synaptophysin-mRuby-WPRE-hGH-pA* (100 nL, PT-1244, 5.65E+12 vg/mL, BrainVTA, China) were injected into the unilateral mPFC of WT mice. Here, mRuby and mGFP were used as marker to show the projection terminals from mPFC to PVT. Further, mRuby could trace the axons from mPFC neurons in the PVT (“Syn^+^” was used in the statistical graph).

For retrograde tracing, the *CTB-555* (80 nL, CTB-02, 1 μg/μL, BrainVTA, China) was injected into the PVT of WT mice. After 1-week (retrograde tracing) and 3/4-week (anterograde tracing) transfection, male mice were perfused with 0.9% saline, followed by 4% PFA and then images of the *CTB-555* or synaptophysin signals were visualized to assess the virus-injected positions. Mice with missed injections were excluded from the study.

### Conditioned place preference (CPP)

The CPP was performed in the TopScan3D CPP apparatus (CleverSys, USA), which is constructed of two distinct chambers (15 × 15 × 23 cm each) separated by a removable guillotine door. One chamber was composed of walls with vertical stripes and a floor with plaid textures. The other chamber was composed of walls with horizontal stripes and a floor with stripes textures. A standard CPP procedure consisted of three phases: the preconditioning test (Baseline/Pre-Test, day 0), conditioning (CPP-Training, day 1-8), and post-conditioning test (CPP-Test, day 9). The mice were habituated at CPP apparatus for 45 min per day from 2 days before Pre-Test. On day 0, Pre-Test (preconditioning test) was assessed by placing the mice in a random chamber of the CPP apparatus and allowing them to explore all two chambers freely for 15 min. After Pre-Test, METH CPP-Training was performed. Based on the Pre-Test, using the non-preferred chamber as drug-paired chamber and the preferred chambers as non-drug-paired chamber. During CPP-Training (conditioning), on day 1, 3, 5 and 7, METH group were intraperitoneally injected with METH (0.1 mg/mL) and immediately restricted to the drug-paired chamber for 45 min, and on day 2, 4, 6 and 8, saline (0.2 mL) was intraperitoneally injected and immediately restricted to the non-drug-paired chamber for 45 min. Saline group were intraperitoneally injected with saline (0.2 mL) throughout the course, and were limited to one side of the chamber on day 1, 3, 5 and 7, and are limited to the other side of the chamber on day 2, 4, 6 and 8. During CPP-Test (post-conditioning test), mice were placed in the chamber of the CPP apparatus and allowing them to explore the two chambers freely without any drug treatment for 15 min. The CPP score is the time spent in drug-paired chamber minus that in non-drug-paired chamber, and the ΔCPP score is the Test score minus the Pre-Test score.

### Immunofluorescence

The male mice were deeply anesthetized with isoflurane (RWD, China) and sequentially perfused with 0.9% saline and 4% paraformaldehyde (PFA). The brains were removed and post-fixed in 4% PFA at 4 °C overnight, then transferred to 30%(w/v) sucrose. The coronal brain sections (30 μm) were continuously sectioned by a cryostat freezing microtome (Leica, Germany) and used for immunofluorescence. The sections were washed in phosphate-buffered saline (PBS) 3 times (10 min each time) and incubated in 0.3% (v/v) Triton X-100 for 30 min, blocked with 5% donkey serum for 90 min at room temperature, and incubated overnight at 4 °C with the following primary antibodies: rabbit anti-c-Fos (1:2000, RRID: AB_2247211, Cell Signaling Technology, USA), mouse anti-NeuN (1:800, RRID: AB_2298772, Millipore, USA), mouse anti-CaMKIIα (1:100, RRID: AB_626789, Santa Cruz, USA), rabbit anti-CaMKIIα (1:200, RRID: AB_305050, Abcam, USA), guinea pig anti-c-Fos (1:3000, RRID: AB_2905595, Synaptic system, Germany), rabbit anti-vGluT2 (1:200, RRID: AB_2799805, Cell Signaling Technology, USA), followed by the corresponding fluorophore-conjugated secondary antibodies for 90 min at room temperature. The following secondary antibodies were used: Alexa Fluor 555-labeled donkey anti-rabbit secondary antibody (1:500, RRID: AB_162543, Invitrogen, USA), Alexa Fluor 488-labeled donkey anti-rabbit (1:500, RRID: AB_2762833, Invitrogen, USA), Fluor 555-labeled donkey anti-mouse secondary antibody (1:500, RRID: AB_2762848, Invitrogen, USA), Fluor 350-labeled donkey anti-mouse secondary antibody (1:500, RRID: AB_2534100, Invitrogen, USA), Fluor 680-labeled donkey anti-rabbit secondary antibody (1:500, RRID: AB_2762836, Invitrogen, USA), Alexa Fluor 488-labeled donkey anti-guinea pig (1:500, RRID: AB_2736871, Abcam, UK). Fluorescence signals were visualized using a Leica DM8I THUNDER Imager 3D Tissue microscope (Leica, Germany) or Leica TCS SP8 Laser Scanning Confocal microscope (Leica, Germany).

### Designer receptors exclusively activated by designer drugs (*DREADDs*)

Male mice were anesthetized with 2% isoflurane in oxygen and were fixed in a stereotactic frame (RWD, China). A heating pad was used to maintain the core body temperature of the animals at 37 °C. The *rAAV2/9-CaMKIIα-hM4D(Gi)-mCherry-WPRE-hGH-pA* (100 nL, PT-0050, 4.81E+12 vg/mL, BrainVTA, China) was injected into the PVT (AP - 1.46 mm, ML 0 mm, DV - 2.95 mm) at a rate of 20 nL/min. After surgery, mice were maintained at home cage about 3 weeks for recovery. Here, the clozapine N-oxide (CNO) was used to modulate the activity of virus-transfected neurons, saline being used as vehicle control. Mice with missed injections were excluded from the study.

For regulation of mPFC-PVT pathway, the *rAAV2/9-CaMKIIα-hM4D(Gi)-mCherry-WPRE-hGH-pA* (200 nL, PT-0050, 4.81E+12 vg/mL, BrainVTA, China) was injected bilaterally into the mPFC of WT mice at a rate of 40 nL/min. The cannula (cat. #62204, O.D. 0.21 mm, RWD, China) was placed 100 μm above the PVT. After surgery, mice were maintained at home cage about 3 weeks for recovery. For all cannula mice, CNO was infused via infusion cannulas at a flow rate of 100 nL/min by a syringe pump (RWD, China). A mixture of S-AMPA (AMPA receptor agonist, 0.125 μg/μL, 250 nL, MCE, USA) and CNO (1 mM, 250 nL, Selleckchem, USA), or mixture of GNE-5729 (GluN2A agonist, 1 μM, 250 nL, MCE, USA) and CNO (1 mM, 250 nL, Selleckchem, USA) was infused via cannulas at a flow rate of 100 nL/min by syringe pump (RWD, China). For the diffusion of the drug, the infusion cannulas were kept in place for 5 min before being replaced with dummy cannulas. Each mixture is injected 5 min before CPP-Test.

### Patch-clamp electrophysiology

Male mice were deeply anesthetized with isoflurane (RWD, China) and perfused with ice-cold cutting solution (in mM: 92 N-methyl-D-glutamine, 2.5 KCl, 1.2 NaH_2_PO_4_, 30 NaHCO_3_, 20 HEPES, 0.5 CaCl_2_, 10 MgSO_4_, 25 glucose, 5 sodium ascorbate, 2 thiourea, and 3 sodium pyruvate, PH adjusted to 7.3 with HCl, osmolarity 305, and saturated with 95% O_2_/5% CO_2_) 26. Slices containing the mPFC or PVT were cut at a 200 μm thickness using a vibratome in 4 °C cutting solution. The slices were transferred to 37 °C cutting solution and kept for ~9 min, then transferred to oxygenated holding solution (in mM: 86 NaCl, 2.5 KCl, 1.2 NaH_2_PO_4_, 35 NaHCO_3_, 20 HEPES, 2 CaCl_2_, 1 MgSO_4_, 25 glucose, 5 sodium ascorbate, 2 thiourea, and 33 sodium pyruvate, PH adjusted to 7.3, osmolarity 305, and saturated with 95% O_2_/5% CO_2_) to allow for recovery at room temperature at least 60 min before recordings. The spike frequency of action potentials (AP) and spontaneous AP (sAP) in neurons were recorded. During electrophysiological recordings, the brain slice was continuously perfused with oxygenated artificial CSF (aCSF, in mM: 119 NaCl, 2.5 KCl, 1 NaH_2_PO_4_, 1.3 MgCl_2_, 2.5 CaCl_2_, 26.2 NaHCO_3_, and 11 glucose, 290 mOsm, saturated with 95% O_2_/5% CO_2_) maintained at 31 °C by a solution heater (TC-324C, Warner Instruments, USA).

Loose-patch microelectrodes were filled with aCSF, and access resistance was maintained at 20-50 MΩ throughout the experiment. Recordings were performed under current-clamp mode with 0 holding current. Whole-cell current-clamp microelectrodes (3-5 MΩ) were filled with K^+^-based internal solution (in mM: 130 K-methane sulfonate, 20 KCl, 10 HEPES, 0.4 EGTA, 2.0 MgCl_2_, 2.5 MgATP, 0.25 Na_3_GTP, 7.5 Na_2_-phosphocreatine, 1 L-glutathione, PH 7.25-7.30; 290 mOsm), current-step protocols were run (step, 25 pA; action potential range, 0-150 pA) at -60 mV. In the receptor screening experiment, SCH-23390 (dopamine 1-like receptor antagonist, 10 μm), NBQX (AMPA receptor antagonist, 10 µM), TCN-201 (GluN2A receptor antagonist, 3 µM), RO-256981 (GluN2B receptor antagonist, 1 µM) was dissolved in oxygenated aCSF to block the specific receptors.

Whole-cell voltage-clamp microelectrodes (3-5 MΩ) were filled with Cs^+^-based internal solution (in mM: 130 CsMeSO_4_, 10 NaCl, 10 EGTA, 4 Mg-ATP, 0.3 Na-GTP, 10 HEPES, PH 7.25-7.30; 290 mOsm), and voltage-clamp protocols were run at -70 mV. The spontaneous excitatory postsynaptic currents (sEPSC) were recorded in voltage-clamp mode at a holding potential of -70 mV. The sEPSC baseline was recorded 5 min after breaking in. The miniature excitatory postsynaptic currents (mEPSC) were recorded at a holding potential of -70 mV in presence of tetrodotoxin (TTX, 1 μM). The amplitude and frequency of the sEPSC and the mEPSC were analyzed.

To verify the synaptic connectivity between mPFC and PVT, *rAAV2/9-CaMKIIα-hChR2(H134R)-mCherry-WPRE-hGH-pA* (200 nL, PT-0297, 5.45E+12 vg/mL, BrainVTA, China) were injected into mPFC, CaMKII-positive neurons in the mPFC were identified by mCherry. The PVT neurons were recorded in voltage clamp. Optical stimulation (473 nm, 5 mW, 20 Hz) was applied on the mPFC ChR2-expressed terminals in the PVT. TTX (1 μM), 4-aminopyridine (4-AP, 100 µM) and TCN-201 (3 µM) were used in the recording aCSF solution to verify whether the mPFC-PVT has a monosynaptic connection. To obtain the paired pulse ratio (PPR), evoked EPSC were elicited by two focal light stimulations (pulse duration of 2 ms, interval of 100 ms). The light stimulations were applied through QAXK-LASTER-473 nm (ThinkerTech, China).

All signals were filtered at 4 kHz, amplified at 5 × using a MultiClamp 700B amplifier (Molecular Devices, USA) and digitized at 10 kHz with a Digidata 1550B analog-to-digital converter (Molecular Devices, USA). All data were analyzed with Clampfit 10.6 software (Molecular Devices, USA).

### Optogenetics

A 593 nm yellow light laser (CLFY-LASEK, China) was used to deliver light pulses to the brain through fiber optic cables (200 μm outer diameter, 0.37 numerical aperture, Inper, China), which was firmly attached to implanted optic fibers by a waveform generator (Inper, China). The power intensity of the laser at the optic fiber terminals with an optical power meter (SANWA, Japan) and calibrated to 10-12 mW at the tip.

In the CPP-Test experiment, the *rAAV2/9-CaMKIIα-eNpHR3.0-mCherry-WPRE-hGH-pA* (200 nL, PT-0009, 5.17E+12 vg/mL, BrainVTA, China) or* rAAV2/9-CaMKIIα-mCherry-WPRE-hGH-pA* (200 nL, PT-0108, 5.29E+12 vg/mL, BrainVTA, China) was injected bilaterally into the mPFC (AP + 1.94 mm, ML ± 0.50 mm, DV - 2.25 mm). The optical fiber (200 μm outer diameter, 0.37 numerical aperture, Inper, China) was placed 100 μm above the PVT. For inhibition of the mPFC-PVT pathway, a constant laser (593 nm, 10 mW, 15 min) was delivered 27-30.

During the patch clamp recording of AP induced by current injection (100 pA for all cells), the delivery of light was set to OFF (500 ms) - ON (1 s) - OFF (500 ms). During recording spontaneous action potentials (sAP), the delivery of light was set to OFF (5 s) - ON (10 s) - OFF (5 s).

### EEG-EMG recordings and analysis

Here, the cohort 12 mice and cohort S3 mice were underwent polysomnographic recordings, which being implanted with EEG-EMG electrodes 31. Among 32 initially used mice, 5 mice were excluded due to missed viral transfections, and 3 mice were excluded due to wrong EEG/EMG electrode implantation. Finally, n = 6 mice per group was subsequent to the analysis. To monitor EEG activity, four small holes were drilled in the frontal and lateral parietal region, and four stainless miniature screws were carefully inserted into the holes to the cortex. EMG electrodes with wire leads were placed between the neck musculature. The EEG-EMG apparatus was affixed to the skull with dental cement. After EEG-EMG electrodes implantation, mice were allowed to recover within individual recording chambers.

The EEG-EMG signals were amplified (Pinnacle 8020-SL, USA) and band-pass filtered (EEG: 0.1-50 Hz, EMG: 70-250 Hz). The signal recording was obtained using the Pinnacle Sirenia acquisition suite (Pinnacle Technology Inc, USA). The polytrophic recording signal was automatically analyzed based on spectral signatures of EEG/EMG waveforms by using the sleep analysis software (Pinnacle Sirenia® Sleep Pro, USA). Wake was defined as desynchronized, low-amplitude EEG rhythms and elevated EMG activity with phasic bursts. NREM sleep was defined as synchronized, high amplitude and low frequency (0.5-4 Hz, delta) EEG activity and lower rhythmic EMG activity. REM sleep was defined as containing a pronounced theta (4-10 Hz) rhythm with nearly no EMG activity.

For regulation of PVT, the *rAAV2/9-CaMKIIα-hM4D(Gi)-mCherry-WPRE-hGH-pA* (100 nL, PT-0050, 4.81E+12 vg/mL, BrainVTA, China) was injected into the PVT of WT mice (AP - 1.46 mm, ML 0 mm, DV - 2.95 mm) at a rate of 20 nL/min. For regulation of mPFC-PVT pathway, the *rAAV2/9-EF1α-DIO-hM4D(Gi)-EGFP-WPRE-hGH-pA* (200 nL, PT-0987, 5.08E+12 vg/mL, BrainVTA, China) was bilaterally injected into the mPFC at a rate of 40 nL/min, and the *rAAV2/R-CaMKIIα-CRE-mCherry-WPRE-hGH-pA* (100 nL, PT-1731, 5.86E+12 vg/mL, BrainVTA, China) were injected into the PVT of WT mice at a rate of 20 nL/min. EEG-EMG electrodes were implanted at the same time of the virus injection. After surgery, mice were maintained at home cage for recovery. After 3-week recovery, the mice were injected with saline (0.2 mL, i.p.) at zeitgeber time 12 (ZT 12, at 19:00) and EEG-EMG signals were collected for 24 h. Then the same mice were also injected with CNO (2 mg/kg, i.p., Selleckchem, USA) at zeitgeber time 12 (ZT 12, at 19:00) and EEG-EMG signals were collected for 24 h. To determine the effects of inhibition of the PVT or mPFC-PVT pathway on sleep-wake states, EEG-EMG power spectra were analyzed during 24 h after CNO or saline injection. The mice used to record sleep-wake states are used here only.

### Data analysis

Statistical analysis was carried out using GraphPad Prism 8.0.2 software. All data are presented as the Mean ± Standard Error of the Mean (SEM). The data were analyzed by unpaired Student's *t*-test, paired Student's *t*-test, One-way Repeated Measures (RM) Analysis of Variance (ANOVA) with Sidak's multiple comparisons test, and Two-way ANOVA with Sidak's multiple comparisons test which appropriate. All statistical significance was set as* p* < 0.05.

## Results

### PVT^Glu^ neurons mediates the acquisition of METH CPP at the stage of retrieval of METH-conditioned context

**Figure 1:**
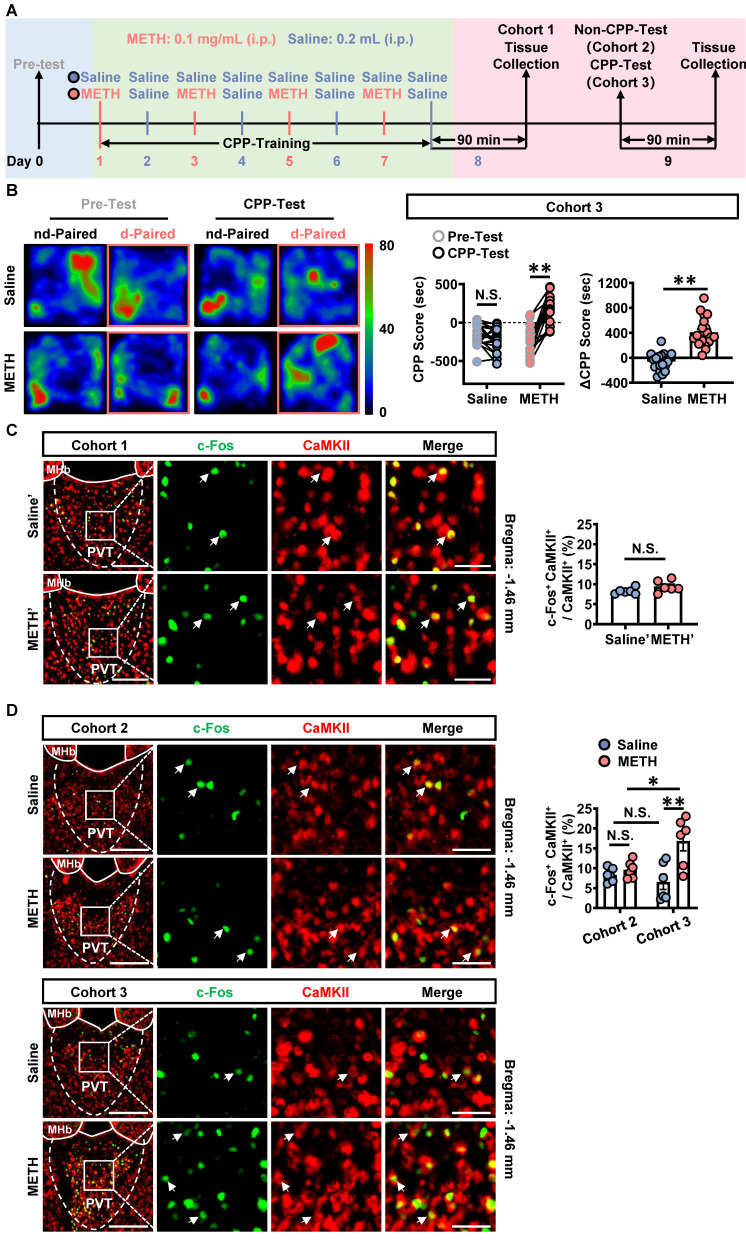
** The glutamatergic neurons of PVT are activated during CPP-Test.** (**A**) Experimental design and timeline of cohort 1, cohort 2 and cohort 3 mice. (**B**) Heatmap of spent duration by mice in CPP apparatus and CPP score analysis of cohort 3 mice. Left panel, Representative heatmap. Middle panel, CPP score during the Pre-Test and CPP-Test. Two-way ANOVA with Sidak's multiple comparisons test. n = 20 mice per group. CPP-Test of Saline group, t = 1.4350, *p* = 0.2935 vs corresponding Pre-Test. CPP-Test of METH group, t = 9.7360, *p* < 0.0001 vs corresponding Pre-Test. Right panel, ΔCPP Score (CPP-Test score minus Pre-Test score). Two-tailed unpaired t test. n = 20 mice per group. t = 7.8990, *p* < 0.0001. (**C**) Immunofluorescence for c-Fos/CaMKII in the PVT of cohort 1 mice. Two-tailed unpaired t test. n = 6 mice per group. t = 1.8220, *p* = 0.0985. Scale bar, 200 μm / 50 μm. (**D**) Immunofluorescence for c-Fos/CaMKII in the PVT of cohort 2 mice and cohort 3 mice. Two-way ANOVA with Sidak's multiple comparisons test. n = 6 mice per group. F _(1, 20)_ = 7.2120,* p* = 0.0142. METH group in cohort 2 mice, t = 0.5616, *p* = 0.9946 vs Saline group in cohort 2 mice. METH group in cohort 3 mice, t = 4.3590, *p* = 0.0018 vs Saline group in cohort 3 mice. Saline group in cohort 3 mice, t = 0.7361, *p* = 0.9779 vs Saline group in cohort 2 mice. METH group in cohort 3 mice, t = 3.062, *p* = 0.0364 vs METH group in cohort 2 mice. Scale bar, 200 μm / 50 μm.

To assess the acquisition of METH-preferred behavior, CPP procedure was performed in male mice (Figure 1A). The mice of cohort 1, cohort 2 and cohort 3 were subjected to CPP-Training (day 1-8), a process that encoding the association of METH with the chamber context. On day 8, the brains of cohort 1 mice were collected at 90 min after the last CPP-Training, forming groups of saline' and METH'. The brains of cohort 2 mice were collected on day 9 without CPP-Test. The brains of cohort 3 mice were collected on day 9 at 90 min after CPP-Test, a process that reflecting the retrieval of METH-conditioned chamber context.

As shown in Figure 1B, METH mice exhibited a higher CPP-Test score than their corresponding Pre-Test score, but not the case for saline mice. Further, the ΔCPP score was much higher in METH mice than that in saline mice. There was no difference in the total distance between the two groups of mice (Figure S1A). These results indicate that METH mice acquired CPP.

The most population neurons in PVT are glutamatergic neurons 32, 33. Here, the c-Fos and CaMKII were respectively used as markers to label the activated neurons and the glutamatergic neurons. As shown in Figure 1C, there was no difference in the percentage of c-Fos and CaMKII double positive neurons in the CaMKII-positive neurons of PVT (glutamatergic neurons of PVT, PVT^Glu^) between saline' mice and METH' mice. As shown in Figure 1D, the percentage of c-Fos and CaMKII double positive neurons in the PVT^Glu^ of cohort 3 mice was higher in the METH group than that in saline group, which performed CPP-Test (Figure 1D). While there was no difference in the percentage of c-Fos and CaMKII double positive neurons in the PVT^Glu^ of cohort 2 mice between saline mice and METH mice, which not performed CPP-Test. These results indicate that PVT^Glu^ is only activated in response to CPP-Test rather than the CPP-Training procedure.

To determine the role of PVT^Glu^ in the acquisition of METH CPP, the virus of *rAAV2/9-CaMKIIα-hM4D(Gi)-mCherry* was injected into PVT in METH mice of cohort S1. CNO was used to inhibit PVT^Glu^ neurons, and saline was used as vehicle control, forming METH-CNO group and METH-Veh group.

In Experiment S1 (Figure S2A-B), CNO was intraperitoneally injected 30 min prior to METH treatment during the period of CPP-Training. As shown in Figure S2C, approximately 90% PVT^Glu^ neurons were transfected with virus. Compared with that of METH-Veh mice, CNO treatment attenuated the percentage of c-Fos and CaMKII double positive neurons in the PVT^Glu^ of METH-CNO mice (Figure S2D). When compared to the corresponding Pre-Test score, METH increased the CPP score both in METH-Veh mice and METH-CNO mice (Figure S2E). There were no significant differences in ΔCPP score (Figure S2E) and total distance (Figure S2F) between METH-Veh mice and METH-CNO mice. These results indicate that inhibition of PVT^Glu^ neurons during the period of CPP-Training has no effects on the acquisition of METH CPP.

**Figure 2:**
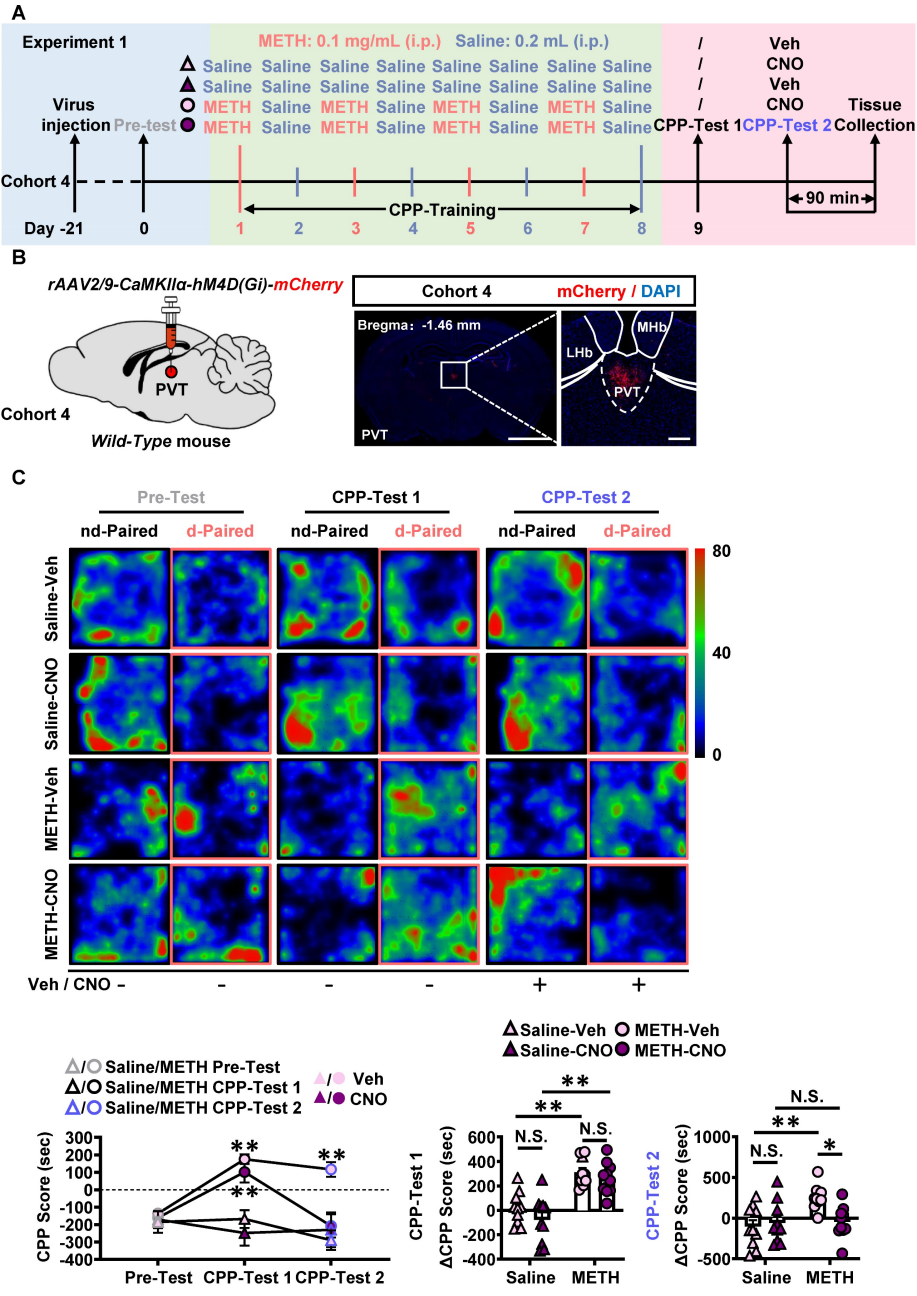
** Chemogenetic inhibition of PVT glutamatergic neurons during CPP-Test disrupts the acquisition of METH CPP.** (**A**) Experimental design and timeline of cohort 4 mice. (**B**) Schematic diagram of viral transfection in WT mice and representative image of *rAAV2/9-CaMKIIα-hM4D(Gi)-mCherry* injection sites in the PVT of cohort 4 mice. Scale bar, 2 mm / 200 μm. (**C**) Heatmap of spent duration by mice in CPP apparatus and CPP analysis of cohort 4 mice. Upper panel, Representative heatmap. Lower panel, CPP analysis. CPP score, Two-way ANOVA with Sidak's multiple comparisons test. n = 10 mice per group. F _(6, 72)_ = 8.0160,* p* < 0.0001. CPP-Test 1 of Saline-Veh group, t = 0.4156, *p* = 0.9695 vs corresponding Pre-Test. CPP-Test 1 of Saline-CNO group, t = 1.2880, *p* = 0.5434 vs corresponding Pre-Test. CPP-Test 1 of METH-Veh group, t = 8.7730, *p* < 0.0001 vs corresponding Pre-Test. CPP-Test 1 of METH-CNO group, t = 6.3800, *p* = 0.0004 vs corresponding Pre-Test. CPP-Test 2 of Saline-Veh group, t = 1.3230, *p* = 0.5228 vs corresponding Pre-Test. CPP-Test 2 of Saline-CNO group, t = 0.7347, *p* = 0.8604 vs corresponding Pre-Test. CPP-Test 2 of METH-Veh group, t = 5.5190, *p* = 0.0011 vs corresponding Pre-Test. CPP-Test 2 of METH-CNO group, t = 0.7646, *p* = 0.8461 vs corresponding Pre-Test. ΔCPP Score of CPP-Test 1, Two-way ANOVA with Sidak's multiple comparisons test. n = 10 mice per group. F _(1, 36)_ = 0.2480,* p* = 0.6215. Saline-CNO group, t = 1.4690, *p* = 0.6242 vs Saline-Veh group. METH-CNO group, t = 0.7648, *p* = 0.9721 vs METH-Veh group. METH-Veh group, t = 4.5630, *p* = 0.0003 vs Saline-Veh group. METH-CNO group, t = 5.2680, *p* < 0.0001 vs Saline-CNO group. ΔCPP Score of CPP-Test 2, Two-way ANOVA with Sidak's multiple comparisons test. n = 10 mice per group. F _(1, 36)_ = 6.4190,* p* = 0.0158. Saline-CNO group, t = 0.4677, *p* = 0.9979 vs Saline-Veh group. METH-CNO group, t = 3.1150, *p* = 0.0214 vs METH-Veh group. METH-Veh group, t = 3.7070, *p* = 0.0042 vs Saline-Veh group. METH-CNO group, t = 0.1241, *p* > 0.9999 vs Saline-CNO group.

In Experiment 1 (Figure 2A-B), no injections were given before CPP-Test 1, while CNO or vehicle injections were given 30 min prior to CPP-Test 2 in cohort 4 mice. As shown in Figure S3A, about 90% PVT^Glu^ neurons were transfected with virus within PVT. As shown in Figure S3B, CNO treatment induced a lower percentage of c-Fos-positive neurons in virus-transfected PVT CaMKII-positive neurons of METH-CNO mice, when compared to that in that of METH-Veh mice. Compared with Saline-Veh mice, METH-Veh mice showed a higher percentage of c-Fos-positive neurons in virus-transfected PVT CaMKII-positive neurons. In CPP-Test 1 (Figure 2C), compared to corresponding Pre-Test, METH mice exhibited a higher CPP score, but saline mice showed similar score, indicating an acquired CPP in METH mice. Compared to Saline-CNO group, the ΔCPP score was much higher in METH-CNO mice. There was no difference in the total distance between the four groups of mice (Figure S3C). In CPP-Test 2 (Figure 2C), when compared to the corresponding vehicle controls, CNO treatment did not affect CPP score in Saline-CNO mice, but attenuated the CPP score in METH-CNO mice. Compared to Saline-Veh mice, the ΔCPP score was much higher in METH-Veh mice. While, the ΔCPP score was lower in METH-CNO mice than that in METH-Veh mice. There was no difference in the total distance between the four groups of mice (Figure S3C). These results showed that the inhibition of PVT^Glu^ neurons only once before CPP-Test could disrupt the acquired METH CPP.

Taken together, these findings suggest that PVT^Glu^ neurons mediates the acquisition of METH CPP at the stage of retrieval of METH-conditioned context, rather than by encoding the association of METH and chamber context.

### GluN2A subunit and AMPA involves in the activation of PVT^Glu^ neurons in METH mice

**Figure 3:**
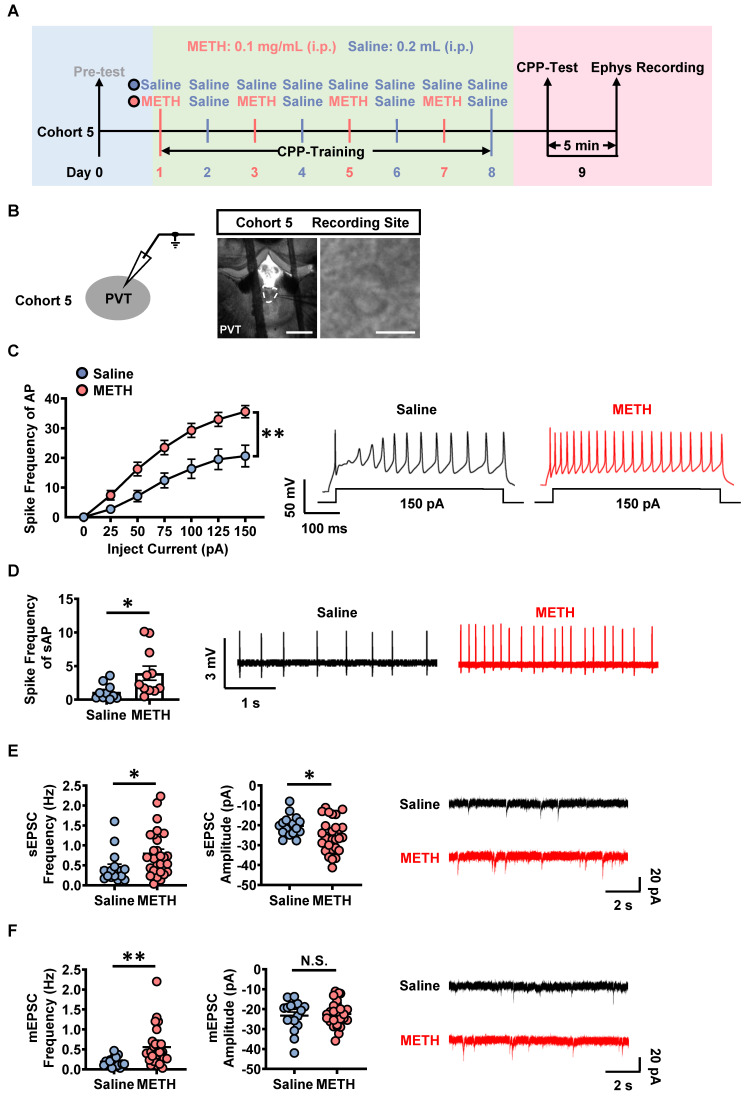
** The activation of PVT neurons in METH mice.** (**A**) Experimental design and timeline of cohort 5 mice. (**B**) Representative images of whole-cell current-clamp recording in the PVT of cohort 5 mice. Scale bar, 200 μm / 50 μm. (**C**) The spike frequency of AP of PVT neurons under whole-cell current-clamp configuration and sample traces for the spike frequency of AP following current injection in Saline mice and METH mice (cohort 5 mice). Two-way ANOVA with Sidak's multiple comparisons test. Saline group, n = 20 cells from 6 mice. METH group, n = 19 cells from 6 mice. F _(6, 222)_ = 7.9050,* p* < 0.0001. (**D**) The spike frequency of sAP of PVT neurons under loose-patch configuration and sample traces for the spike frequency of sAP in Saline mice and METH mice (cohort 5 mice). Two-tailed unpaired t test. Saline group, n = 10 cells from 6 mice. METH group, n = 11 cells from 6 mice. t = 2.3910, *p* = 0.0273. (**E**) The spontaneous EPSC (sEPSC) in the PVT neurons of cohort 5 mice. Quantification of the frequency (left) and amplitude (middle) of sEPSC and sample traces of sEPSC (right). Two-tailed unpaired t test. Saline group, n = 17 cells from 6 mice. METH group, n = 27 cells from 6 mice. Frequency, t = 2.2810, *p* = 0.0277. Amplitude, t = 2.1840, *p* = 0.0346. (**F**) The miniature EPSC (mEPSC) in the PVT neurons of cohort 5 mice. Quantification of the frequency (left) and amplitude (middle) of mEPSC and sample traces of mEPSC (right). Two-tailed unpaired t test. Saline group, n = 17 cells from 6 mice. METH group, n = 27 cells from 6 mice. Frequency, t = 3.0750, *p* = 0.0037. Amplitude, t = 0.4158, *p* = 0.6797.

On day 9, cohort 5 mice were subjected to patch-clamp recordings after CPP-Test (Figure 3A-B). Previously, most studies demonstrate that PVT receives dense glutamatergic and dopaminergic projections 21, 28, 34. Therefore, we screened which kind of PVT projections was involved in METH CPP by using corresponding antagonists. The SCH-23390, RO-256981, TCN-201 and NBQX were used as specific antagonists for dopamine 1-like receptors (D1R), GluN2B- and GluN2A-containing NMDA receptors, AMPA glutamate receptors, respectively.

As shown in Figure 3C, the spike frequency of action potentials (AP) was higher in PVT slices of METH mice than that of saline mice under whole-cell recording, so as to the spike frequency of spontaneous AP (sAP) in slices under loose-patch recording (Figure 3D). As shown in Figure 3E and Figure 3F, when compared to PVT slices of saline mice, the frequency and amplitude of the sEPSC, as well as the frequency of mEPSC were increased in that of METH mice, but no difference in the amplitude of mEPSC.

**Figure 4:**
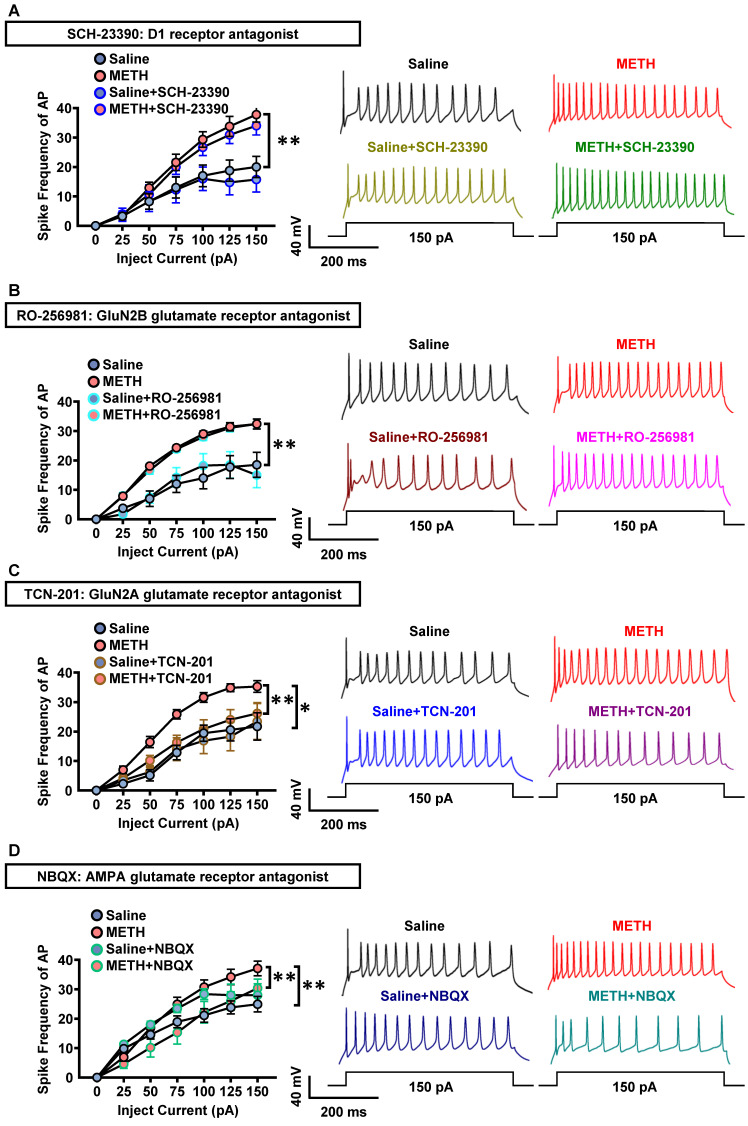
** GluN2A and AMPA receptors involves in the triggered PVT neurons in METH mice.** (**A**) The spike frequency of AP of PVT neurons (cohort 5 mice) under whole-cell current-clamp configuration following current injection in the absence and presence of SCH-23390 on the same cell and sample traces (right panel). Two-way ANOVA with Sidak's multiple comparisons test. Saline group, n = 8 cells from 3 mice. Saline+SCH-23390 group, n = 8 cells from 3 mice. METH group, n = 9 cells from 4 mice. METH+SCH-23390 group, n = 9 cells from 4 mice. METH group, F _(6, 105)_ = 3.6380,* p* = 0.0026 vs Saline group. METH+SCH-23390 group, F _(6, 56)_ = 0.3077, *p* = 0.9304 vs METH group. Saline+SCH-23390 group, F _(6, 49)_ = 0.4146, *p* = 0.8658 vs Saline group. (**B**) The spike frequency of AP of PVT neurons (cohort 5 mice) under whole-cell current-clamp configuration following current injection in the absence and presence of RO-256981 on the same cell and sample traces (right panel). Two-way ANOVA with Sidak's multiple comparisons test. Saline group, n = 8 cells from 4 mice. Saline+RO-256981 group, n = 8 cells from 4 mice. METH group, n = 26 cells from 6 mice. METH+RO-256981 group, n = 26 cells from 6 mice. METH group, F _(6, 224)_ = 5.0230, *p* < 0.0001 vs Saline group. METH+RO-256981 group, F _(6, 175)_ = 0.3038, *p* = 0.9343 vs METH group. Saline+RO-256981 group, F _(6,49)_ = 0.5073, *p* = 0.7998 vs Saline group. (**C**) The spike frequency of AP of PVT neurons (cohort 5 mice) under whole-cell current-clamp configuration following current injection in the absence and presence of TCN-201 on the same cell and sample traces (right panel). Two-way ANOVA with Sidak's multiple comparisons test. Saline group, n = 7 cells from 4 mice. Saline+TCN-201 group, n = 7 cells from 4 mice. METH group, n = 14 cells from 6 mice. METH+TCN-201 group, n = 14 cells from 6 mice. METH group, F _(6, 133)_ = 3.4870, *p* = 0.0031 vs Saline group. METH+TCN-201 group, F _(6, 91)_ = 2.3140, *p* = 0.0400 vs METH group. Saline+TCN-201 group, F _(6, 42)_ = 0.8165, *p* = 0.5633 vs Saline group. (**D**) The spike frequency of AP of PVT neurons (cohort 5 mice) under whole-cell current-clamp configuration following current injection in the absence and presence of NBQX on the same cell and sample traces (right panel). Two-way ANOVA with Sidak's multiple comparisons test. n = 11 cells from 6 mice per group. METH group, F _(6, 140)_ = 4.1820,* p* = 0.0007 vs Saline group. METH+NBQX group, F _(6, 70)_ = 3.9340, *p* = 0.0019 vs METH group. Saline+NBQX group, F _(6, 70)_ = 0.7585, *p* = 0.6049 vs Saline group.

As shown in Figure 4A-D, in PVT slices of METH mice, the spike frequency of AP in PVT neurons did not significantly change after incubation with SCH-23390 (Figure 4A) or RO-256981 (Figure 4B), but decreased after incubation with TCN-201 (Figure 4C) or NBQX (Figure 4D). In PVT slices of saline mice, SCH-23390 (Figure 4A), RO-256981 (Figure 4B), TCN-201 (Figure 4C) and NBQX (Figure 4D) did not change the spike frequency of AP in PVT neurons.

These results indicate that GluN2A subunit and AMPA, but not D1R or GluN2B subunit, take part in the activation of PVT neurons of METH mice, suggesting the potential contribution of glutamatergic innervation on PVT to the acquisition of METH CPP.

### PVT receives the glutamatergic innervations from mPFC

**Figure 5:**
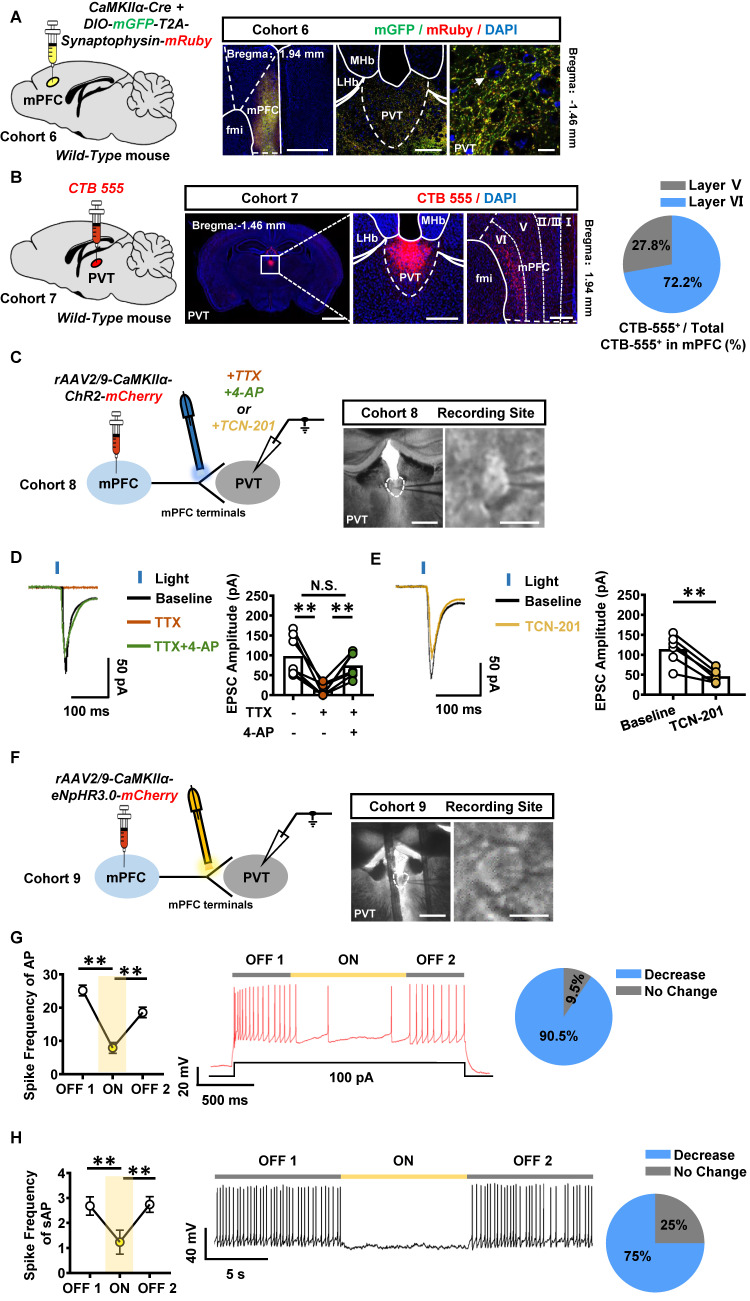
** The mPFC and PVT exist structural and functional connections.** (**A**) Schematic diagram of viral transfection in WT mice (left) and representative image of the mixed of *rAAV2/9-CaMKIIα-Cre-WPRE* and* rAAV2/9-hSyn-DIO-mGFP-T2A-Synaptophysin-mRuby-WPRE* injection sites in the mPFC and expression around the PVT neurons of cohort 6 mice (right). Scale bar, 500 μm / 200 μm / 50 μm. (**B**) Schematic diagram of *CTB-555* in WT mice (left) and representative image of the *CTB-555* injection sites in the PVT and expression within the mPFC of cohort 7 mice (middle) and the proportion of *CTB-555* at layer V and VI of the total *CTB-555* in mPFC (right). Scale bar, 2 mm / 200 μm / 50 μm. (**C**) Schematic diagram and representative image of whole-cell voltage-clamp recording site in the PVT of cohort 8 mice. Scale bar, 200 μm / 50 μm. (**D**) Example traces (left) and quantification of the amplitude of EPSC (right) following TTX and 4-AP treatment during optogenetic activation of mPFC-PVT terminals in cohort 8 mice. Two-tailed paired t test. n = 7 cells from 4 mice. TTX, t = 4.8120, *p* = 0.0030 vs Baseline. TTX+4-AP, t = 4.9340, *p* = 0.0026 vs TTX. TTX+4-AP, t = 1.2360, *p* = 0.2628 vs Baseline. (**E**) Example traces (left) and quantification of the amplitude of EPSC (right) following TCN-201 treatment during optogenetic activation of mPFC-PVT terminals in cohort 8 mice. Two-tailed paired t test. n = 6 cells from 4 mice. t = 6.9870, *p* = 0.0009. (**F**) Schematic diagram and representative image of whole-cell current-clamp recording site in the PVT of cohort 9 mice. Scale bar, 200 μm / 50 μm. (**G**) The spike frequency of AP in PVT neurons under whole-cell current-clamp configuration and sample traces for the spike frequency of AP following optogenetic stimulation in naive mice (cohort 9 mice). Left, Quantification of AP spike frequency. Middle, sample traces. Right, percentage of AP changes in the PVT neurons (19 out of 21 cells from 4 mice, 90.5%), no change (2 out of 21 cells from 4 mice, 9.5%). One-way ANOVA with Sidak's multiple comparisons test. n = 21 cells from 4 mice per group. F _(1.181, 23.62)_ = 52.36, *p* < 0.0001. ON, t = 9.0470, *p* < 0.0001 vs OFF 1; t = 5.0190, *p* = 0.0002 vs OFF 2. (**H**) The spike frequency of sAP in PVT neurons under loose-patch configuration and sample traces for the spike frequency of sAP following optogenetic stimulation in naive mice (cohort 9 mice). Left, quantification of sAP frequency. Middle, sample trace. Right, percentage of sAP changes in PVT neurons. Light-induced decreased firing (12 out of 15 cells from 4 mice, 75%), no change (3 out of 15 cells from 4 mice, 25%). One-way ANOVA with Sidak's multiple comparisons test. n = 15 cells from 4 mice per group. F _(1.654, 23.15)_ = 19.9800, *p* < 0.0001. ON, t = 5.8060, *p* = 0.0001 vs OFF 1; t = 4.6370, *p* = 0.0012 vs OFF 2.

The anatomical architecture and physiological function of mPFC-PVT pathway was explored in naïve male mice of cohort 6 and cohort 7. An anterograde virus of CaMKII promoter-expressed Cre (*rAAV2/9-CaMKIIα-Cre-WPRE-hGH-pA*) and Cre-dependent mGFP and mRuby (*rAAV2/9-hSyn-DIO-mGFP-T2A-Synaptophysin-mRuby-WPRE-hGH-pA*) were unilaterally injected into mPFC to trace its glutamatergic efferent projections in cohort 6 mice (Figure 5A). A retrograde tracer of *CTB-555* was injected into PVT of cohort 7 mice (Figure 5B). As shown in Figure 5A-B, synaptophysin fluorescence was highly expressed around the PVT neurons, and *CTB-555* was expressed in the layer V and VI of mPFC, with 27.8% neurons being located in the layer V, and 72.2% neurons being located in the layer VI of mPFC.

As shown in Figure S4A-B, the whole-cell patch clamp on PVT neurons, combined with optogenetic activation of ChR2-expressing PVT-projecting mPFC neurons, were recorded in cohort S2 mice. As shown in Figure S4C, compared to that of saline mice, the paired pulse ratio (PPR) was increased in PVT neurons of METH mice, suggesting a change in presynaptic glutamatergic terminals on the mPFC-PVT pathway during the acquisition of METH CPP.

To further assess the physiological innervations of mPFC glutamatergic projections on PVT neurons, the *rAAV2/9-CaMKIIα-ChR2-mCherry* was injected into mPFC of WT cohort 8 mice, and light-evoked EPSC were recorded in PVT by stimulating ChR2-expressing terminals with blue light (Figure 5C). Administration of sodium channel blocker tetrodotoxin (TTX) blocked the EPSC, whereas the combination of potassium channel blocker 4-aminopyridine (4-AP) rescued them, suggesting that mPFC glutamatergic neurons form monosynaptic connections with downstream PVT neurons (Figure 5D). Additionally, GluN2A subunit antagonist TCN-201 partially blocked the light-evoked EPSC (Figure 5E). In addition, an optogenetic virus of *rAAV2/9-CaMKIIα-eNpHR3.0-mCherry-WPRE-hGH-pA* was injected into mPFC of WT cohort 9 mice, the spike frequency was recorded in mPFC and PVT neurons *in vitro* (Figure S5A and Figure 5F). As shown in Figure S5B, the mPFC neurons that transfected with the virus showed a decrease in spike frequency of AP by turning-on the yellow lights, and then mPFC neurons showed an increase in spike frequency of AP when turning-off the yellow lights. As shown in Figure 5G-H, when modulating mPFC glutamatergic terminals within PVT, 90.5% of PVT neurons showed a decrease in spike frequency of AP by lights-on, and an increase in spike frequency of AP when lights-off. For the spike frequency of sAP, 75% of PVT neurons showed a decrease by lights-on, and an increase in spike frequency of AP when lights-off.

Taken together, these results suggest the mPFC sends direct glutamatergic projection to PVT, which can positively regulate PVT neuronal activity, forming mPFC-PVT glutamatergic pathway, of which exhibited altered electrophysiological properties in presynaptic glutamatergic terminals during the acquisition of METH CPP.

### The mPFC-PVT glutamatergic pathway mediates the acquisition of METH CPP

**Figure 6:**
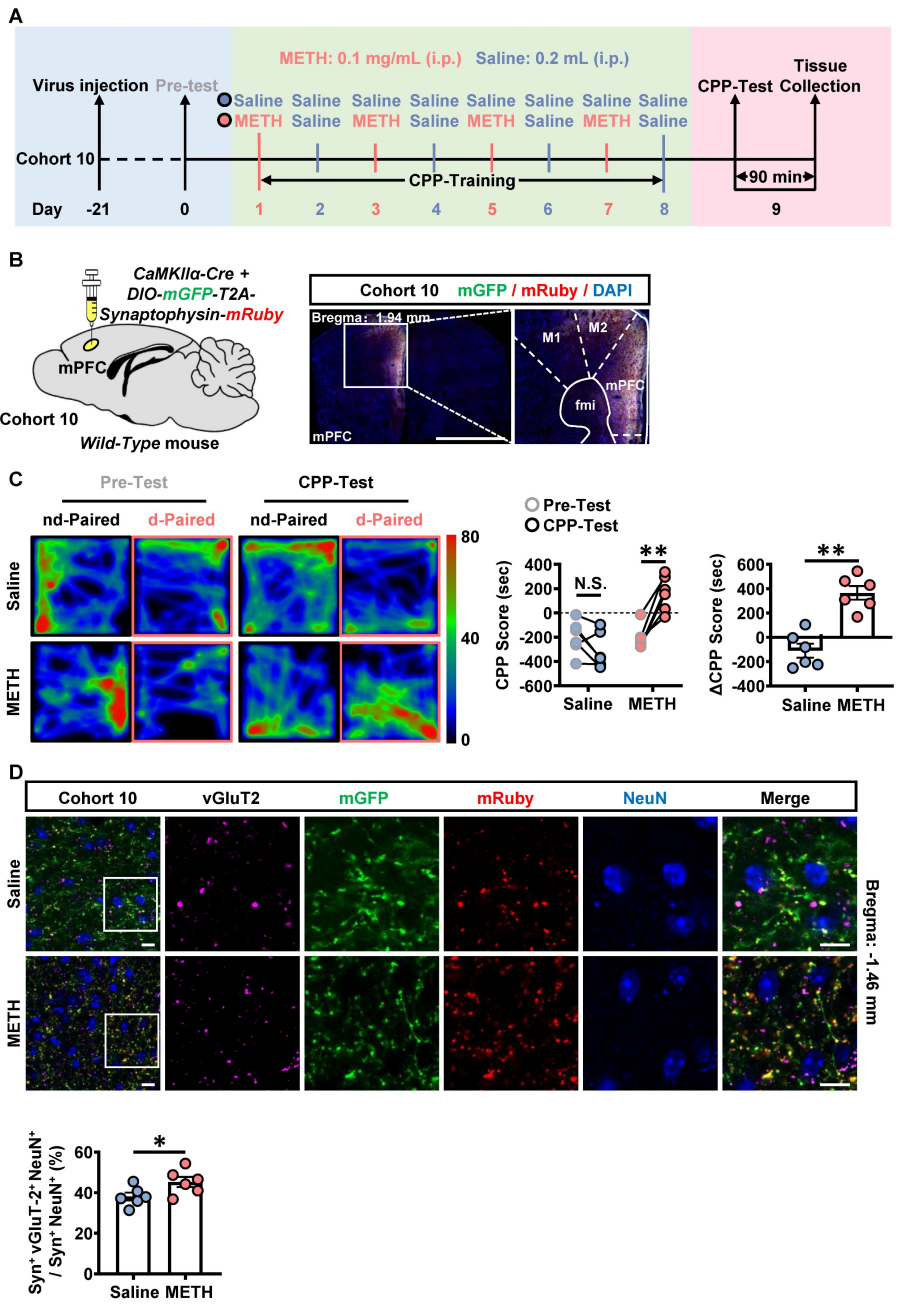
** The mPFC glutamatergic terminals within PVT are activated during the acquisition of METH CPP.** (**A**) Experimental design and timeline of cohort 10 mice. (**B**) Schematic diagram of viral transfection in WT mice and representative image of the mixed of *rAAV2/9-CaMKIIα-Cre-WPRE* and* rAAV2/9-hSyn-DIO-mGFP-T2A-Synaptophysin-mRuby-WPRE* injection sites in the mPFC of cohort 10 mice. Scale bar, 2 mm / 200 μm. (**C**) Heatmap of spent duration by mice in CPP apparatus and CPP analysis of cohort 10 mice. Left panel, Representative heatmap. Middle panel, CPP Score during the Pre-Test and CPP-Test. Two-way ANOVA with Sidak's multiple comparisons test. n = 6 mice per group. CPP-Test of Saline group, t = 1.9290, *p* = 0.1583 vs corresponding Pre-Test. CPP-Test of METH group, t = 6.4220, *p* = 0.0002 vs corresponding Pre-Test. Right panel, ΔCPP Score (CPP-Test score minus Pre-Test score). Two-tailed unpaired t test. n = 6 mice per group. t = 5.9050, *p* = 0.0002. (**D**) Immunofluorescence for vGluT2/Synaptophysin/NeuN in the PVT following METH-induced CPP-Test in cohort 10 mice. Two-tailed unpaired t test. n = 6 mice per group. t = 2.2780, *p* = 0.0459. Scale bar, 50 μm / 50 μm.

The neuronal activity in response to METH CPP-Test was examined in the mPFC of cohort 3 mice and in the mPFC-PVT pathway of cohort 10 mice. As shown in Figure S6A, when compared with that of saline mice, the percentage of c-Fos-positive neurons of mPFC was significantly increased at both II/III layers and V/VI layers in METH mice during the acquisition of METH CPP. As shown in Figure 6A-B, the mPFC glutamatergic terminals were marked in the PVT by the virus injecting into the mPFC (*rAAV2/9-CaMKIIα-Cre-WPRE* and *rAAV2/9-hSyn-DIO-mGFP-T2A-Synaptophysin-mRuby-WPRE-hGH-pA*) 35-37. The vGluT2 was used to label pre-synaptic synaptic vesicles, and in part could represent the glutamatergic terminals 38. The Syn^+^ indicate the overlapped influence of mGFP and mRuby. As shown in Figure 6C, there was a higher CPP-Test score than the corresponding Pre-Test score in METH mice, and ΔCPP score were at higher level in METH mice than that in saline mice, with no difference in total distance between the two groups (Figure S6B), indicating an acquired CPP in METH mice. Compared to saline mice, there was an increased number of vGluT2-positive virus-transfected mPFC glutamatergic terminals to wrap around PVT neurons in METH mice (Figure 6D), indicating more activated mPFC glutamatergic terminals within PVT during the acquisition of METH CPP.

**Figure 7:**
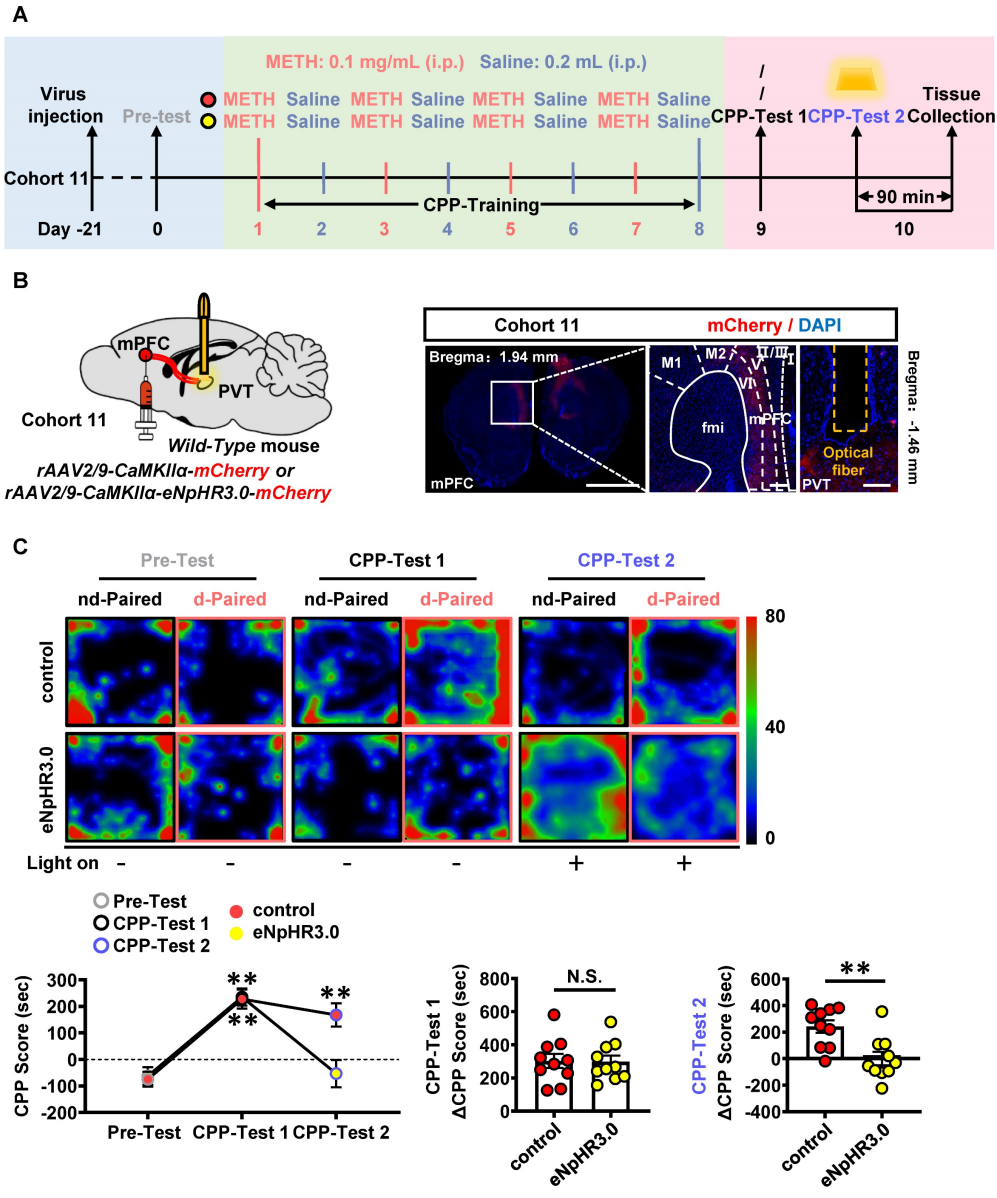
** Suppressing mPFC-PVT pathway disrupts the METH-acquired CPP.** (**A**) Experimental design and timeline of cohort 11 mice. (**B**) Schematic diagram of viral transfection and optical fiber implantation in WT mice (cohort 11 mice, Left panel). Representative image of *rAAV2/9-CaMKIIα-eNpHR3.0-mCherry* injection sites in the mPFC and optical fiber embedding sites in PVT of WT mice (cohort 11 mice, Right panel). Scale bar, 2 mm / 200 μm / 200 μm. (**C**) Heatmap of spent duration by mice in CPP apparatus and CPP analysis of cohort 11 mice. Upper panel, Representative heatmap. Lower panel, CPP analysis. CPP score, Two-way ANOVA with Sidak's multiple comparisons test. n = 10 mice per group. F _(2, 36)_ = 9.7810,* p* = 0.0004. CPP-Test 1 of control group, t = 7.0820, *p* = 0.0002 vs corresponding Pre-Test. CPP-Test 1 of *eNpHR3.0* group, t = 8.0810, *p* < 0.0001 vs corresponding Pre-Test. CPP-Test 2 of control group, t = 1.5080, *p* = 0.4196 vs corresponding CPP-Test 1. CPP-Test 2 of *eNpHR3.0* group, t = 7.4360, *p* = 0.0001 vs corresponding CPP-Test 1. ΔCPP Score of CPP-Test 1, Two-tailed unpaired t test. n = 10 mice per group. t = 0.0805, *p* = 0.9367. ΔCPP Score of CPP-Test 2, Two-tailed unpaired t test. n = 10 mice per group. t = 3.5180, *p* = 0.0025.

To examine the role of mPFC-PVT pathway in the acquisition of METH CPP, the optogenetic virus of *rAAV2/9-CaMKIIα-eNpHR3.0-mCherry* was bilaterally injected into mPFC of cohort 11 mice, along with a fiber implanting in the PVT. The virus of *rAAV2/9-CaMKIIα-mCherry* was used as virus control (Figure 7A-B). About 80%~95% CaMKII-positive neurons were transfected with the virus (Figure S7A), and light-on could efficiently decrease the percentage of c-Fos-positive PVT neurons in *eNpHR3.0* group, when compared with virus control mice (Figure S7B). The yellow light kept turning-off during CPP-Test 1. As shown in Figure 7C, METH mice obtained higher CPP scores than their Pre-Test score, indicating an acquired CPP. The yellow light kept turning-on during CPP-Test 2. As shown in Figure 7C, the light-on reduced CPP score in METH mice when compared to that of light-off period, and the ΔCPP score was much lower in *eNpHR3.0* mice than that in virus controls. There was no difference in the total distance between *eNpHR3.0* mice and virus controls (Figure S7C).

Taken together, these results suggest that the glutamatergic projections from mPFC to PVT involves in the acquisition of METH CPP.

### Inhibition of PVT^Glu^ neurons rather than mPFC-PVT glutamatergic pathway affects the wakefulness

The PVT^Glu^ neurons play an important role in the process of wakefulness 31. Modulating PVT carries risk to interference with sleep and arousal cycle 39, 40. As shown in Figure S8A, the virus of *rAAV2/9-CaMKIIα-hM4D(Gi)-mCherry* was injected into PVT of cohort S3 mice, and EEG/EMG were recorded *in vivo*. As shown in Figure S8B-E, compared to vehicle treatment, inhibition of PVT^Glu^ neurons by CNO injection reduced the wakefulness period but increased the NREM period in mice.

**Figure 8:**
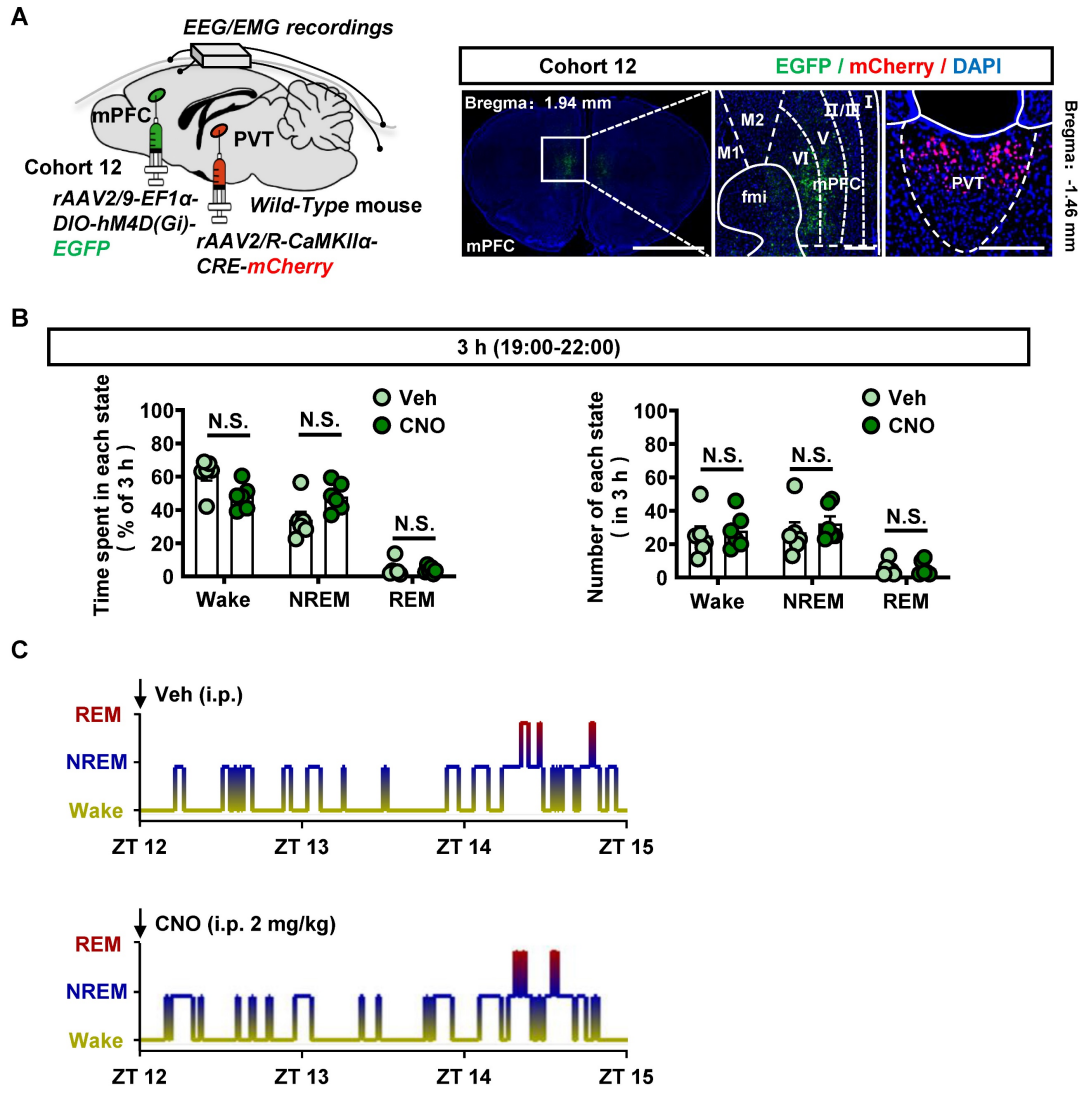
** Suppressing mPFC-PVT pathway has no influence on wakefulness.** (**A**) Schematic diagram of viral transfection in WT mice and representative image of *rAAV2/9-EF1α-DIO-hM4D(Gi)-EGFP* injection sites in the mPFC and *rAAV2/R-CaMKIIα-CRE-mCherry* injection sites in the PVT of cohort 12 mice. Scale bar, 2 mm / 200 μm / 200 μm. (**B**) Percentage of time spent and number in each state within 3 h after injection of saline or CNO in cohort 12 mice. Two-way ANOVA with Sidak's multiple comparisons test. n = 6 mice per group. Time spent, F _(2, 20)_ = 5.8170,* p* = 0.0102. Wake, t = 2.6550, *p* = 0.0740; NREM, t = 2.3710, *p* = 0.1200; REM, t = 0.2355, *p* = 0.9942. Number, F _(2, 20)_ = 0.3393,* p* = 0.7163. Wake, t = 0.4066, *p* = 0.9711; NREM, t = 0.7009, *p* = 0.8755; REM, t = 0.0672, *p* = 0.9999. (**C**) EEG/EMG recording representative hypnograms during 3 h after saline or CNO injection in cohort 12 mice.

Next, to examine the effects of modulating mPFC-PVT pathway on the wakefulness, the virus of *rAAV2/9-EF1α-DIO-hM4D(Gi)-EGFP* were injected into mPFC, and the *rAAV2/R-CaMKIIα-CRE-mCherry* were injected into PVT of cohort 12 mice (Figure 8A)*.* As shown in Figure 8B-C and Figure S9A, inhibition of mPFC-PVT pathway by CNO injection had no influences on the wakefulness period, NREM period and REM period in mice.

Taken together, these results indicate that inhibition of PVT^Glu^ neurons, rather than the inhibition of mPFC-PVT pathway, has risk of interference with the wakefulness.

### The GluN2A subunit involves in the regulatory role of mPFC-PVT pathway in the acquisition of METH CPP

**Figure 9:**
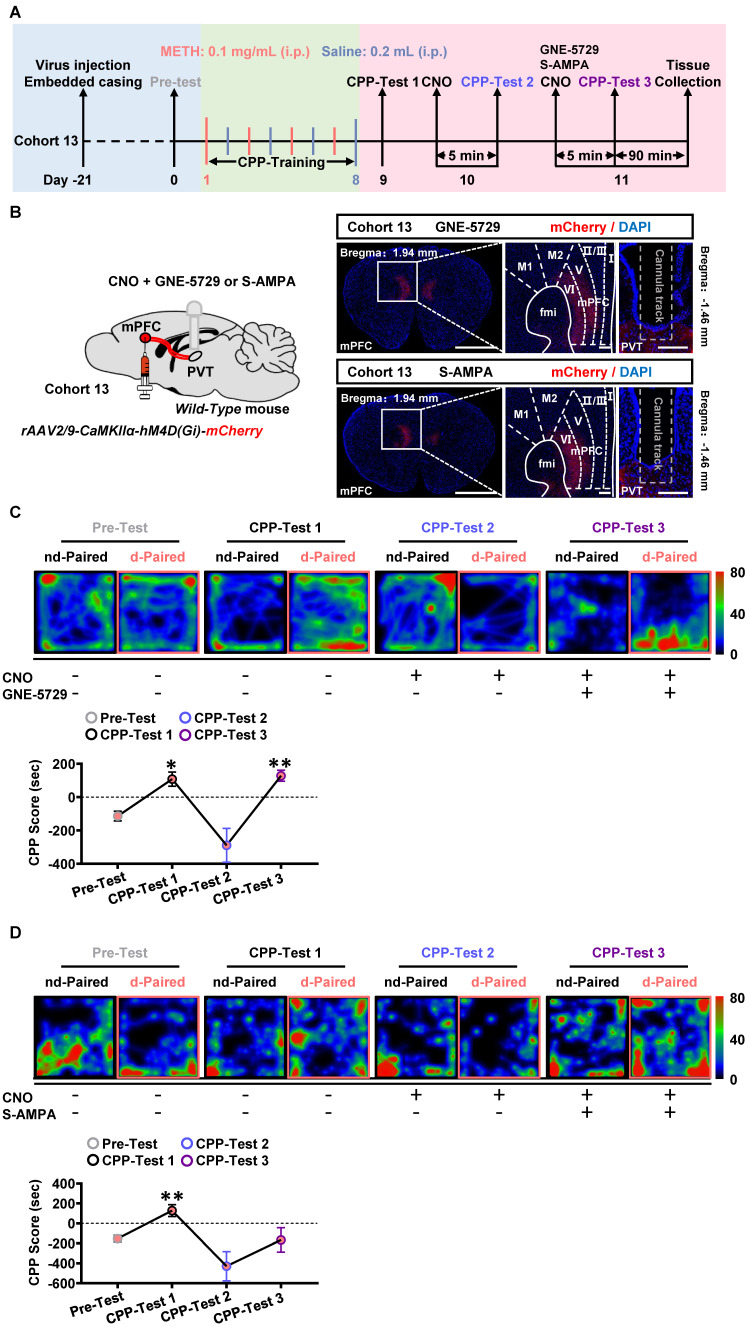
** Postsynaptic GluN2A is a key molecule along mPFC-PVT pathway that involves in the acquisition of METH CPP.** (**A**) Experimental design and timeline of cohort 13 mice. (**B**) Schematic diagram of viral transfection and cannula implantation in WT mice (cohort 13 mice, Left panel). Representative image of *rAAV2/9-CaMKIIα-hM4D(Gi)-mCherry* injection sites in the mPFC and cannula embedding sites in the PVT (cohort 13 mice, Right panel). Scale bar, 2 mm / 200 μm / 200 μm. (**C**) Heatmap of spent duration by mice in CPP apparatus and CPP analysis in GNE-5729 group mice (cohort 13 mice). Upper panel, Representative heatmap. Lower panel, CPP analysis, One-way ANOVA with Sidak's multiple comparisons test. n = 7 mice. F _(1.364, 8.183)_ = 13.2200,* p* = 0.0044. CPP-Test 1, t = 4.4110, *p* = 0.0268 vs Pre-Test. CPP-Test 2, t = 1.7710, *p* = 0.5569 vs Pre-Test. CPP-Test 3, t = 5.9030, *p* = 0.0063 vs Pre-Test. (**D**) Heatmap of spent duration by mice in CPP apparatus and CPP analysis in S-AMPA group mice (cohort 13 mice). Upper panel, Representative heatmap. Lower panel, CPP analysis, One-way ANOVA with Sidak's multiple comparisons test. n = 7 mice. F _(1.922, 11.53)_ = 8.4840,* p* = 0.0057. CPP-Test 1, t = 5.5050, *p* = 0.0090 vs Pre-Test. CPP-Test 2, t = 2.0910, *p* = 0.3994 vs Pre-Test. CPP-Test 3, t = 0.1403, p > 0.9999 vs Pre-Test.

Here, the GNE-5729 and S-AMPA were respectively used as GluN2A agonist and AMPA receptor agonist. As shown in Figure 9A-B, the virus of *rAAV2/9-CaMKIIα-hM4D(Gi)-mCherry* was bilaterally injected into mPFC of cohort 13 mice. Neither CNO nor any agonist was given in the CPP-Test 1. CNO were infused into PVT during CPP-Test 2. A mixture of CNO and GNE-5729 or S-AMPA were injected into the PVT in the CPP-Test 3.

As shown in Figure 9C-D, in CPP-Test 1, CPP score became higher at CPP-Test than that at Pre-Test in METH group, indicating an acquired CPP. In the CPP-Test 2, compared to corresponding Pre-Test score, CPP-Test 2 score was reduced to normal levels by CNO treatment in METH mice, indicating the therapeutic effects of suppressing mPFC-PVT pathway in the acquired CPP. In CPP-Test 3, a mixture of CNO and GNE-5729 or S-AMPA were used to inhibit mPFC glutamatergic terminals and to respectively activate GluN2A or AMPA receptors within the PVT. The mixture of CNO and GNE-5729 infusion reversed the CPP score to a higher level in METH mice, but not changed the CPP score with the mixture of CNO and S-AMPA infusion, indicating that GNE-5729 counteract the amelioration effects of CNO treatment on the acquisition of METH CPP. As shown in Figure S10A, there was no difference in the total distance during the CPP-Test among all groups.

Taken together, these results suggest the regulatory role of mPFC-PVT pathway in the acquisition of METH CPP at least in part mediated by GluN2A subunit in the PVT.

## Discussion

**Figure 10:**
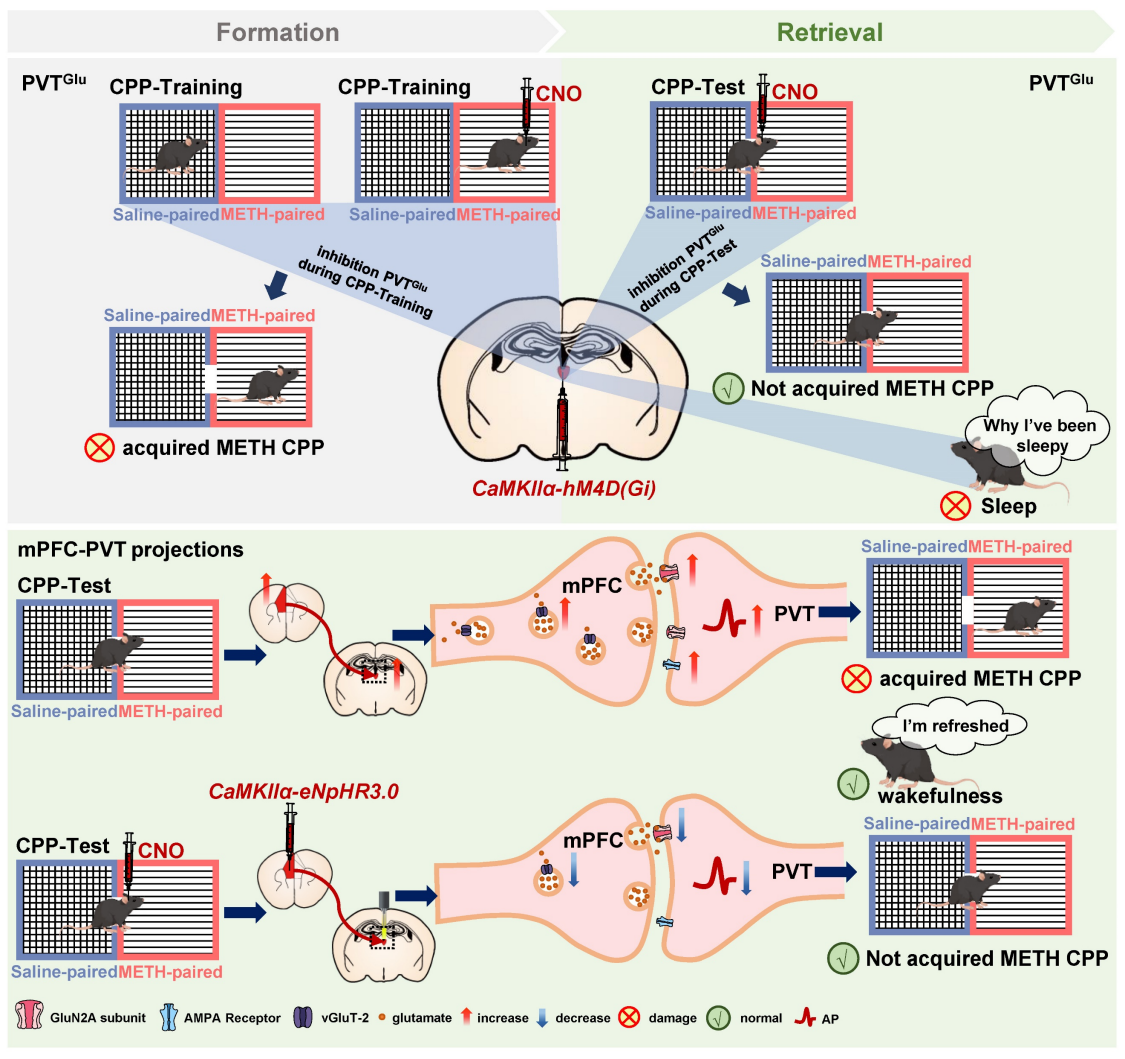
** Schematic diagram of the present study.** METH CPP acquisition activates PVT glutamatergic neurons (PVT^Glu^) and the glutamatergic projections from mPFC to PVT in male mice. Suppressing PVT^Glu^ during the METH CPP-Test rather than the training of METH CPP disrupts the acquisition of METH CPP, while its activity affects wakefulness. However, inhibition of the glutamatergic projections from mPFC to PVT disrupts the METH-acquired CPP without influencing wakefulness. The postsynaptic GluN2A receptor in mPFC-PVT glutamatergic projections may be a promising therapeutic target for mediating the acquisition of METH CPP.

In the current study, consistent with previous studies of cocaine CPP 5, 8, we found that the glutamatergic neurons of PVT (PVT^Glu^) are involved in the regulation of METH CPP acquisition. However, we also found that modulating PVT^Glu^ neurons increases the risk of altering the arousal and sleep rhythms. Therefore, it is necessary to find PVT-based neuronal circuits which can reduce the rewarding effects of METH and avoid affecting wakefulness-related biorhythms. We found that inhibition of glutamatergic projections from mPFC to PVT efficiently disrupts the acquisition of METH CPP, and most importantly, does not affect wakefulness. The GluN2A subunit of NMDA receptor mediates the regulatory role of mPFC-PVT pathway in the development of METH reward (Figure 10).

The PVT has been recognized as an integrative hub for reward and substance abuse 6, 41-43. The CPP procedure is often used to evaluate reward in rodents. As a kind of conditional reflex behavior, the CPP-Training procedure represents an encoding process that links reward (e.g. METH) with a conditioned context (e.g. METH-paired chamber), while the CPP-Test represents a retrieval process of reward memory (releasing). It is still unclear at which stage of CPP procedure that PVT actually involves. Here, we found that the PVT^Glu^ neurons are only triggered by METH-conditioned context, and the inhibition of PVT^Glu^ neurons only once before METH CPP-Test could disrupt the acquisition of METH CPP, indicating an involvement of PVT in the retrieval process of METH reward.

Previous studies including ours showed that mPFC play important roles in METH reward 14-17. In rodents, the mPFC sends dense glutamatergic projections to PVT. Emerging evidence demonstrates that the mPFC-PVT pathway take part in the process of memory retrieval 19, 22, 44. Previous studies reported that suppressing mPFC inputs to the PVT could attenuate cocaine seeking behaviors in rats 20, 45, implying a potential top-down control of mPFC-PVT glutamatergic pathway in METH reward. Here, we found that inhibition of glutamatergic projections from mPFC to PVT blocks the acquisition of METH CPP, and does not alter the wakefulness in mice. Of course, in addition to the mPFC, the PVT also receives projections from the insular cortices, the hippocampal, and other areas 18. In future studies, we will explore the different roles of the glutamatergic projections to PVT from different sources in the METH reward process.

The NMDA receptors and AMPA receptors are the main ionic glutamatergic mediators along the mPFC-PVT pathway. We found that both GluN2A of NMDA receptors and AMPA receptors contribute to the triggered PVT neurons in METH mice. However, only GluN2A agonist could disrupt the therapeutic effects of mPFC-PVT pathway on the acquisition of METH CPP. In this study, we focused on the functional impact of NMDA and AMPA receptor activity in the PVT by employing chemogenetic viral combined pharmacological regulatory strategies. The current results could not fully reflect the postsynaptic GluN2A's role in the mPFC-PVT glutamatergic pathway. In future studies, we will use more spatially restricted recording techniques to explore the potential molecules along the mPFC-PVT pathway that are implicated in the METH reward.

The lack of female mice models is a limitation of the current study. Female mice undergo estrous cycles, which result in dramatic changes during these cycles. The hormonal changes have been shown to affect both neuronal excitability and behavioral outcomes 46-48. In future studies, it is necessary to explore whether there exists gender difference in the regulatory role of the glutamatergic projections from mPFC to PVT on the acquisition of METH CPP.

## Conclusions

PVT^Glu^ is involved in the reward process of METH at the retrieval stage of METH-conditioned context, rather than at the stage of encoding association between METH and context. The glutamatergic projections from mPFC to PVT, especially the GluN2A molecule, may be a promising therapeutic target for reducing METH reward, as there are no significant side effects on wakefulness.

## Supplementary Material

Supplementary figures.

## Abbreviations

aCSF: artificial CSF
CNO: clozapine-N-oxide
CPP: conditioned place preference
DREADDs: designer receptors exclusively activated by designer drugs
Glu: glutamatergic neurons
IACUC: Institutional Animal Care and Use Committee
METH: methamphetamine
mPFC: medial prefrontal cortex
PBS: phosphate-buffered saline
PFA: paraformaldehyde
PVT: paraventricular nucleus of thalamus
WT: wild type
