# Supplementary material for: Glutamatergic pathways from medial prefrontal cortex to paraventricular nucleus of thalamus contribute to the methamphetamine-induced conditioned place preference without affecting wakefulness
Source: Theranostics. 2025 Jan 2;15(5):1822–41. doi: 10.7150/thno.100688 (PMC11780515; doi:10.7150/thno.100688)
Supplement: Supplementary file 1 — Supplementary figures. [file thnov15p1822s1.pdf]

Supplementary figure legends

Figure S1. The glutamatergic neurons of PVT are activated during CPP-Test

(**A**) Total distance traveled in CPP apparatus during CPP-Test of cohort 3 mice. Two-tailed unpaired t test. n = 20 mice per group. t = 1.4370, *p* = 0.1589.

Figure S2. Chemogenetic inhibition of PVT glutamatergic neurons during CPP-Training does not block the acquisition of METH CPP

(**A**) Experimental design and timeline of cohort S1 mice.

(**B**) Schematic diagram of viral transfection in WT mice and representative image of *rAAV2/9-CaMKIIα-hM4D(Gi)-mCherry* injection sites in the PVT of cohort S1 mice. Scale bar, 2 mm / 200 μm.

(**C**) The percentage of virus transfected efficiency in CaMKII-positive neurons of PVT in cohort S1 mice. Two-tailed unpaired t test. n = 4 mice per group. t = 0.3114, *p* = 0.7661. Scale bar, 200 μm / 50 μm.

(**D**) The percentage of c-Fos-positive neurons on *mCherry*-transfected neurons in the PVT of cohort S1 mice. Two-tailed unpaired t test. n = 4 mice per group. t = 3.4940, *p* = 0.0129. Scale bar, 200 μm / 50 μm.

(**E**) Heatmap of spent duration by mice in CPP apparatus and CPP analysis of cohort S1 mice. Left panel, Representative heatmap. Middle panel, CPP score during the Pre-Test and CPP-Test. Two-way ANOVA with Sidak's multiple comparisons test. n = 10 mice per group. CPP-Test of METH-Veh group, t = 6.3140, $p < 0.0001$ vs corresponding Pre-Test. CPP-Test of METH-CNO group, t = 3.6610, *p* = 0.0036 vs corresponding Pre-Test. Right panel, ΔCPP score (CPP-Test score minus Pre-Test score). Two-tailed unpaired t test. n = 10 mice per group. t = 1.8760, *p* = 0.0770.

(**F**) Total distance traveled in CPP apparatus during CPP-Test of cohort S1 mice. Two-tailed unpaired t test. n = 10 mice per group. t = 0.0381, *p* = 0.9700.

Figure S3. Chemogenetic inhibition of PVT glutamatergic neurons during CPP-Test disrupts the acquisition of METH CPP

(**A**) The percentage of virus transfected efficiency in CaMKII-positive neurons of PVT in cohort 4 mice. Two-way ANOVA with Sidak's multiple comparisons test. n = 4 mice per group. $F_{(1, 12)} = 0.0131$, $p = 0.9108$. Saline-CNO group, t = 0.0961, $p > 0.9999$ vs Saline-Veh group. METH-CNO group, t = 0.2580, $p > 0.9999$ vs METH-Veh group. Scale bar, 200 μm / 50 μm.

(**B**) The percentage of c-Fos-positive neurons on *mCherry*-transfected neurons in the PVT of cohort 4 mice. Two-way ANOVA with Sidak's multiple comparisons test. n = 4 mice per group. $F_{(1, 12)} = 5.8370$, $p = 0.0326$. Saline-CNO group, t = 0.0091, $p > 0.9999$ vs Saline-Veh group. METH-CNO group, t = 3.4070, $p = 0.0308$ vs METH-Veh group. METH-Veh group, t = 3.2950, $p = 0.0378$ vs Saline-Veh group. Scale bar, 200 μm / 50 μm.

(**C**) Total distance traveled in CPP apparatus during CPP-Test 1 and CPP-Test 2 of cohort 4 mice. Two-way ANOVA with Sidak's multiple comparisons test. n = 10 mice per group. CPP-Test 1, $F_{(1, 36)} = 0.0078$, $p = 0.9297$. Saline-CNO group, t = 0.6667, $p = 0.9860$ vs Saline-Veh group. METH-CNO group, t = 0.7923, $p = 0.9669$ vs METH-Veh group. METH-Veh group, t = 1.0310, $p = 0.8915$ vs Saline-Veh group. CPP-Test 2, $F_{(1, 36)} = 3.0030$, $p = 0.0917$. Saline-CNO group, t = 0.6742, $p = 0.9852$ vs Saline-Veh group. METH-CNO group, t = 1.7770, $p = 0.4096$ vs METH-Veh group. METH-Veh group, t = 2.5240, $p = 0.0930$ vs Saline-Veh group.

Figure S4. The paired pulse ratio was increased within PVT neurons during the acquisition of METH CPP

(A) Experimental design and timeline of cohort S2 mice.

(B) Schematic diagram and representative image of whole-cell voltage-clamp recording site in the PVT of cohort S2 mice. Scale bar, 200 μm / 50 μm.

(C) Paired pulse ratio (PPR) recordings. Representative traces (left) and quantification of PPR in PVT neurons (right). Two-tailed unpaired t test. n = 13 cells from 3 mice per group. t = 2.2150, $p = 0.0365$.

Figure S5. The spike frequency of AP decreased in mPFC *eNpHR3.0-mCherry*-transfected neurons by turning-on the yellow lights

(**A**) Schematic diagram of viral transfection in WT mice and representative image of whole-cell current-clamp recording site in the mPFC of cohort 9 mice. Scale bar, 200 μm / 50 μm.

(**B**) The spike frequency of AP of mPFC *eNpHR3.0-mCherry*-transfected neurons under whole-cell current-clamp configuration and sample traces for the spike frequency of AP following optogenetic stimulation in naive mice (cohort 9 mice). Left, quantification of AP spike frequency. Middle, sample trace. Right, percentage of AP changes in the mPFC *eNpHR3.0-mCherry*-transfected neurons (25 out of 25 cells from 4 mice, 100%). One-way ANOVA with Sidak's multiple comparisons test. n = 25 cells from 4 mice per group. $F_{(1.206, 28.94)} = 80.0100$, $p < 0.0001$. ON, t = 9.8570, $p < 0.0001$ vs OFF 1; t = 8.4170, $p < 0.0001$ vs OFF 2.

Figure S6. The mPFC glutamatergic neurons are activated during CPP-Test

(**A**) Immunofluorescence for c-Fos/CaMKII in the mPFC layer II/III and layer V and VI following METH-induced CPP-Test of cohort 3 mice. Two-tailed unpaired t test. n = 6 mice per group. Layer II/III, t = 2.4740, $p = 0.0329$. Layer V and VI, t = 5.1660, $p = 0.0004$. Scale bar, 200 μm / 50 μm.

(**B**) Total distance traveled in CPP apparatus during CPP-Test of cohort 10 mice. Two-tailed unpaired t test. n = 6 mice per group. t = 0.6627, $p = 0.5225$.

Figure S7. Suppressing mPFC-PVT pathway disrupts the acquired METH CPP

(**A**) The percentage of virus transfected efficiency in CaMKII-positive neurons of mPFC in cohort 11 mice. Two-tailed unpaired t test. n = 4 mice per group. t = 0.0265, $p = 0.9797$. Scale bar, 200 μm / 50 μm.

(**B**) The percentage of c-Fos-positive neurons on *mCherry*-transfected neurons following optogenetic stimulation in the PVT of cohort 11 mice. Two-tailed unpaired t test. n = 4 mice per group. t = 5.7540, $p = 0.0012$. Scale bar, 200 μm / 50 μm.

(**C**) Total distance traveled in CPP apparatus during CPP-Test 1 and CPP-Test

2 of cohort 11 mice. CPP-Test 1, Two-tailed unpaired t test. n = 10 mice per group. t = 0.7133, p = 0.4848. CPP-Test 2, Two-tailed unpaired t test. n = 10 mice per group. t = 1.9790, p = 0.0633.

Figure S8. Suppression of PVT glutamatergic neurons caused a reduction in wakefulness

(**A**) Schematic diagram of viral transfection in WT mice and representative image of *rAAV2/9-CaMKIIα-hM4D(Gi)-mCherry* injection sites in the PVT of cohort S3 mice. Scale bar, 2 mm / 200 μm.

(**B**) Percentage of time spent and number in each state within 3 h after injection of saline or CNO of cohort S3 mice. Two-way ANOVA with Sidak's multiple comparisons test. n = 6 mice per group. Time spent in each state within 3 h, $F_{(2, 20)}$ = 43.1300, p < 0.0001. Wake, t = 6.8580, p = 0.0008; NREM, t = 6.3610, p = 0.0013; REM, t = 2.0800, p = 0.2154. Number in each state within 3 h, $F_{(2, 20)}$ = 28.8800, p < 0.0001. Wake, t = 3.3330, p = 0.0489; NREM, t = 3.3650, p = 0.0307; REM, t = 0.1832, p = 0.9973.

(**C**) EEG/EMG recording representative hypnograms during 3 h after saline or CNO injection of cohort S3 mice.

(**D**) Heatmap of relative EEG power and example traces of EEG/EMG in different sleep states defined by Sirenia Sleep.

(**E**) Percentage of time spent and number in each state within 24 h、12 h (dark phase) and 12 h (light phase) after injection of saline or CNO in cohort S3 mice. Two-way ANOVA with Sidak's multiple comparisons test. n = 6 mice per group. Time spent in each state within 24 h, $F_{(2, 20)}$ = 26.5500, p < 0.0001. Wake, t = 5.1120, p = 0.0015; NREM, t = 5.2820, p = 0.0011; REM, t = 1.6340, p = 0.3758. Number in each state within 24 h, $F_{(2, 20)}$ = 31.7900, p < 0.0001. Wake, t = 3.2690, p = 0.0261; NREM, t = 3.1800, p = 0.0302; REM, t = 0.1406, p = 0.9987. Time spent in each state within 12 h (dark phase), $F_{(2, 20)}$ = 31.6900, p < 0.0001. Wake, t = 5.6120, p = 0.0009; NREM, t = 5.6990, p = 0.0008; REM, t = 1.2230, p = 0.5956. Number in each state within 12 h (dark phase), $F_{(2, 20)}$ = 28.1100, p < 0.0001. Wake, t = 3.1860, p = 0.0298; NREM, t

= 3.3300, p = 0.0230; REM, t = 0.2447, p = 0.9933. Time spent in each state within 12 h (light phase), $F_{(2, 20)}$ = 0.1017, p = 0.9038. Wake, t = 0.3544, p = 0.9807; NREM, t = 0.2485, p = 0.9931; REM, t = 1.7320, p = 0.3410. Number in each state within 12 h (light phase), $F_{(2, 20)}$ = 12.6600, p = 0.0003. Wake, t = 2.0860, p = 0.1801; NREM, t = 2.1280, p = 0.1686; REM, t = 0.2187, p = 0.9952.

Figure S9. Suppressing mPFC-PVT pathway has no influence on wakefulness

(**A**) Percentage of time spent and number in each state within 24 h、12 h (dark phase) and 12 h (light phase) after injection of saline or CNO in cohort 11 mice. Two-way ANOVA with Sidak's multiple comparisons test. n = 6 mice per group. Time spent in each state within 24 h, $F_{(2, 20)}$ = 2.6210, p = 0.0975. Wake, t = 1.5160, p = 0.4568; NREM, t = 1.7520, p = 0.3580; REM, t = 0.8095, p = 0.8239. Number in each state within 24 h, $F_{(2, 20)}$ = 0.0213, p = 0.9789. Wake, t = 0.2565, p = 0.9925; NREM, t = 0.1834, p = 0.9972; REM, t = 0.5401, p = 0.9366. Time spent in each state within 12 h (dark phase), $F_{(2, 20)}$ = 0.5591, p = 0.5804. Wake, t = 0.7563, p = 0.8486; NREM, t = 0.7479, p = 0.8527; REM, t = 0.3548, p = 0.9805. Number in each state within 12 h (dark phase), $F_{(2, 20)}$ = 0.4269, p = 0.6583. Wake, t = 0.8301, p = 0.7978; NREM, t = 0.9122, p = 0.7487; REM, t = 0.0912, p = 0.9996. Time spent in each state within 12 h (light phase), $F_{(2, 20)}$ = 2.6330, p = 0.0966. Wake, t = 1.5020, p = 0.4368; NREM, t = 1.7740, p = 0.3027; REM, t = 0.8538, p = 0.8036. Number in each state within 12 h (light phase), $F_{(2, 20)}$ = 0.1004, p = 0.9049. Wake, t = 0.5258, p = 0.9421; NREM, t = 0.4109, p = 0.9710; REM, t = 0.7163, p = 0.8695.

Figure S10. Postsynaptic GluN2A is a key molecule along mPFC-PVT pathway that involves in the acquisition of METH CPP

(**A**) Total distance traveled in CPP apparatus of GNE-5729 (Left) and S-AMPA (Right) mice (cohort 13 mice). GNE-5729, One-way ANOVA with Sidak's multiple comparisons test. n = 7 mice. $F_{(2.068, 12.41)}$ = 3.2800, p = 0.0706. CPP-

Test 1, t = 3.2880, $p = 0.0958$ vs Pre-Test. CPP-Test 2, t = 1.0290, $p = 0.9196$ vs Pre-Test. CPP-Test 3, t = 0.3908, $p = 0.9994$ vs Pre-Test. S-AMPA, One-way ANOVA with Sidak's multiple comparisons test. n = 7 mice. $F_{(2.028, 12.17)} = 3.6800$, $p = 0.0558$. CPP-Test 1, t = 0.1041, $p > 0.9999$ vs Pre-Test. CPP-Test 2, t = 3.1040, $p = 0.1196$ vs Pre-Test. CPP-Test 3, t = 1.9430, $p = 0.4688$ vs Pre-Test.

Figure S1

A

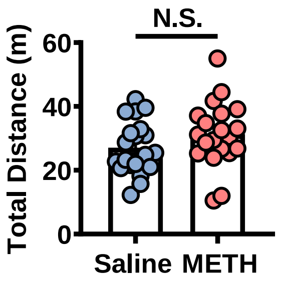

Figure S2

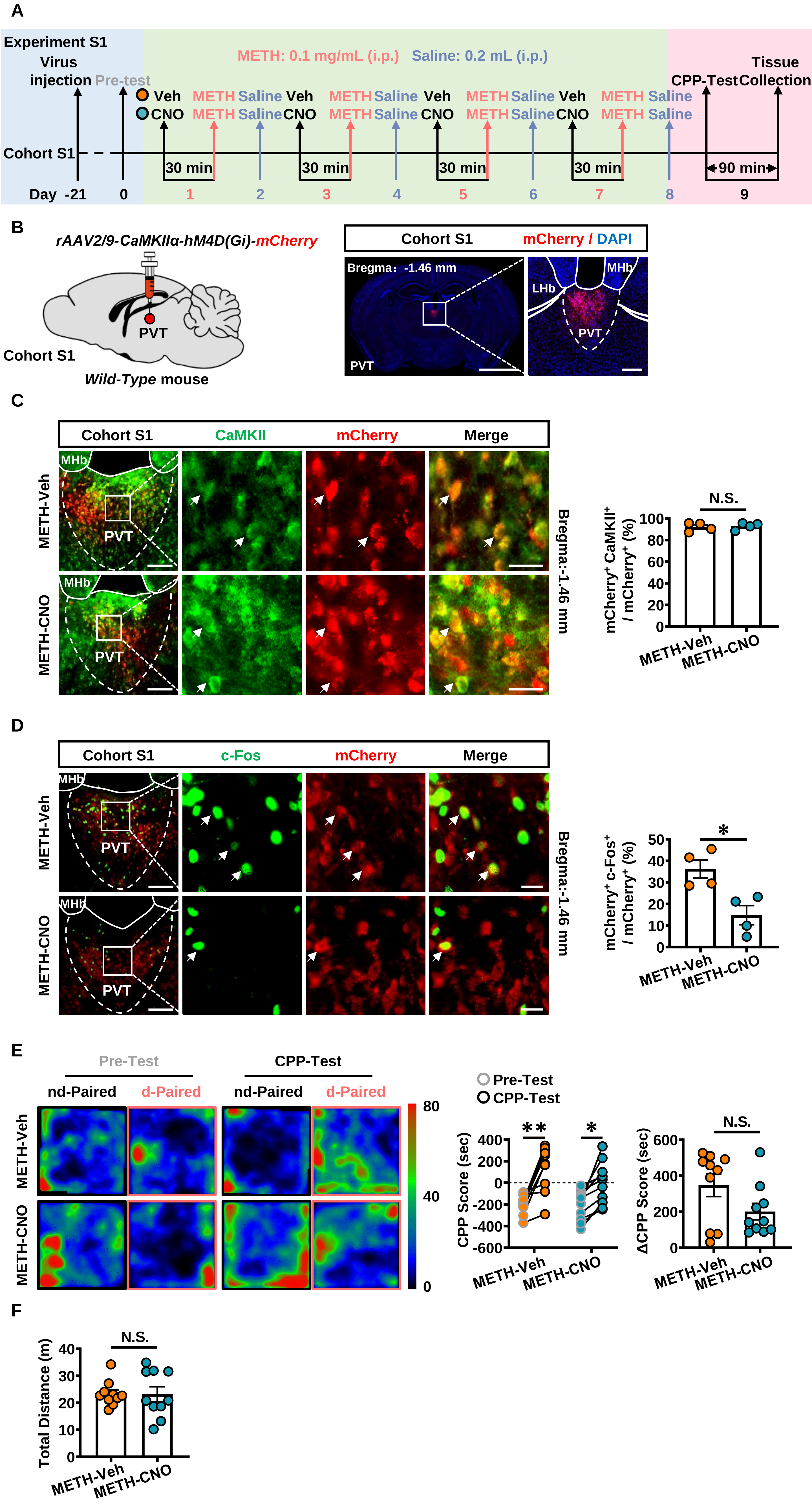
A
Experiment S1
Virus injection
Pre-test
METH: 0.1 mg/mL (i.p.)
Saline: 0.2 mL (i.p.)
Veh
CNO
METH Saline Veh
METH Saline CNO
CPP-Test
Tissue Collection
Cohort S1
30 min
90 min
Day -21 0 1 2 3 4 5 6 7 8 9
B
rAAV2/9-CaMKIIα-hM4D(Gi)-mCherry
PVT
Cohort S1
Wild-Type mouse
mCherry / DAPI
Bregma: -1.46 mm
MHb
LHb
C
Cohort S1
CaMKII
mCherry
Merge
METH-Veh
METH-CNO
Bregma:-1.46 mm
N.S.
mCherry⁺ CaMKII⁺ / mCherry⁺ (%)
D
c-Fos
mCherry⁺ c-Fos⁺ / mCherry⁺ (%)
*
E
Pre-Test
CPP-Test
nd-Paired
d-Paired
80
40
0
Pre-Test
CPP-Test
CPP Score (sec)
**
*
ΔCPP Score (sec)
F
Total Distance (m)
N.S.

Figure S3

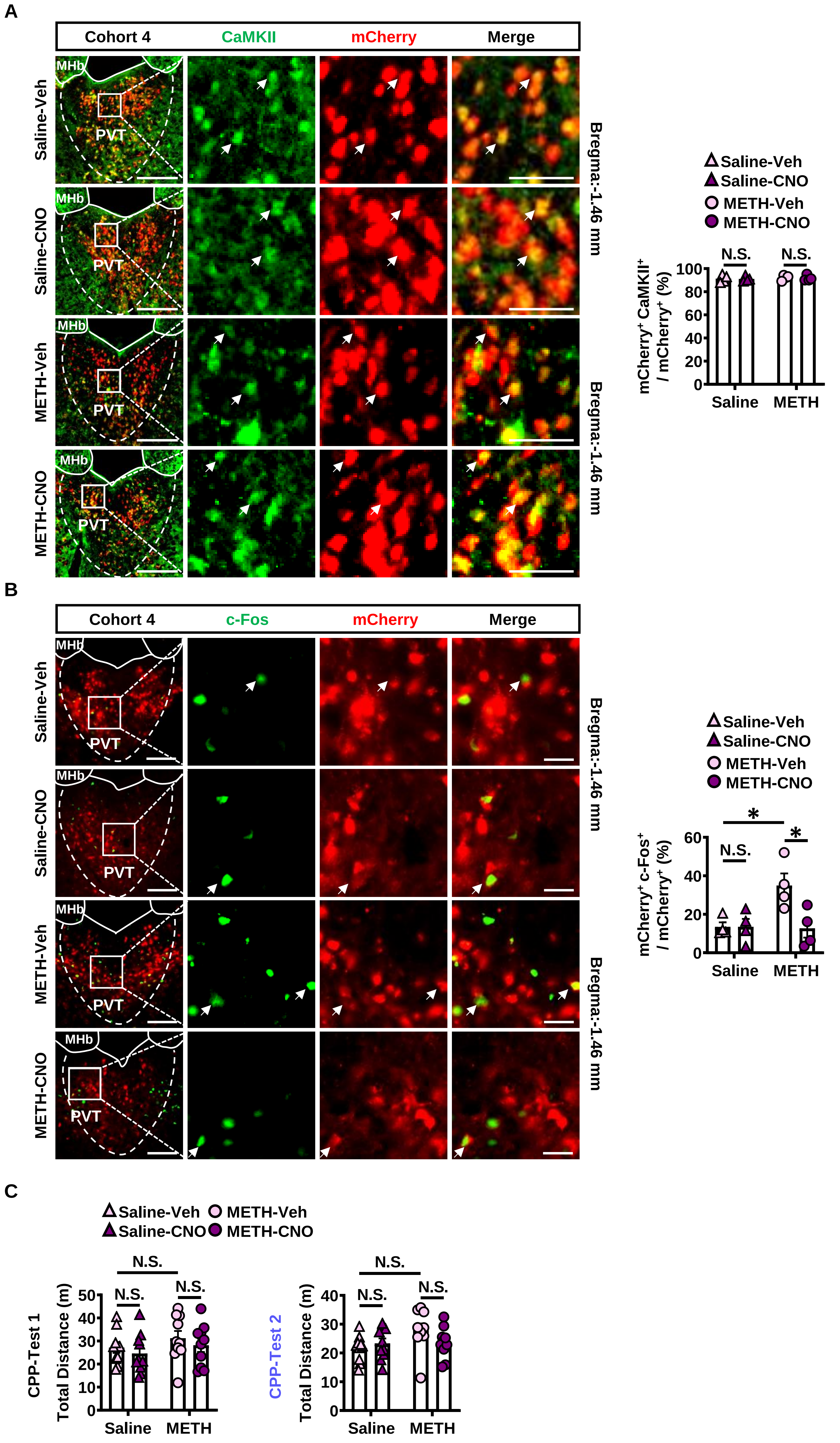
A
Cohort 4
CaMKII
mCherry
Merge
Saline-Veh
Saline-CNO
METH-Veh
METH-CNO
MHb
PVT
Bregma:-1.46 mm
Saline-Veh
Saline-CNO
METH-Veh
METH-CNO
N.S.
N.S.
mCherry+ CaMKII+ / mCherry+ (%)
0
20
40
60
80
100
Saline
METH
B
Cohort 4
c-Fos
mCherry
Merge
Saline-Veh
Saline-CNO
METH-Veh
METH-CNO
MHb
PVT
Bregma:-1.46 mm
Saline-Veh
Saline-CNO
METH-Veh
METH-CNO
*
N.S.
*
mCherry+ c-Fos+ / mCherry+ (%)
0
20
40
60
Saline
METH
C
Saline-Veh
Saline-CNO
METH-Veh
METH-CNO
N.S.
N.S.
N.S.
CPP-Test 1
Total Distance (m)
0
10
20
30
40
50
Saline
METH
N.S.
N.S.
N.S.
CPP-Test 2
Total Distance (m)
0
10
20
30
40
Saline
METH

Figure S4

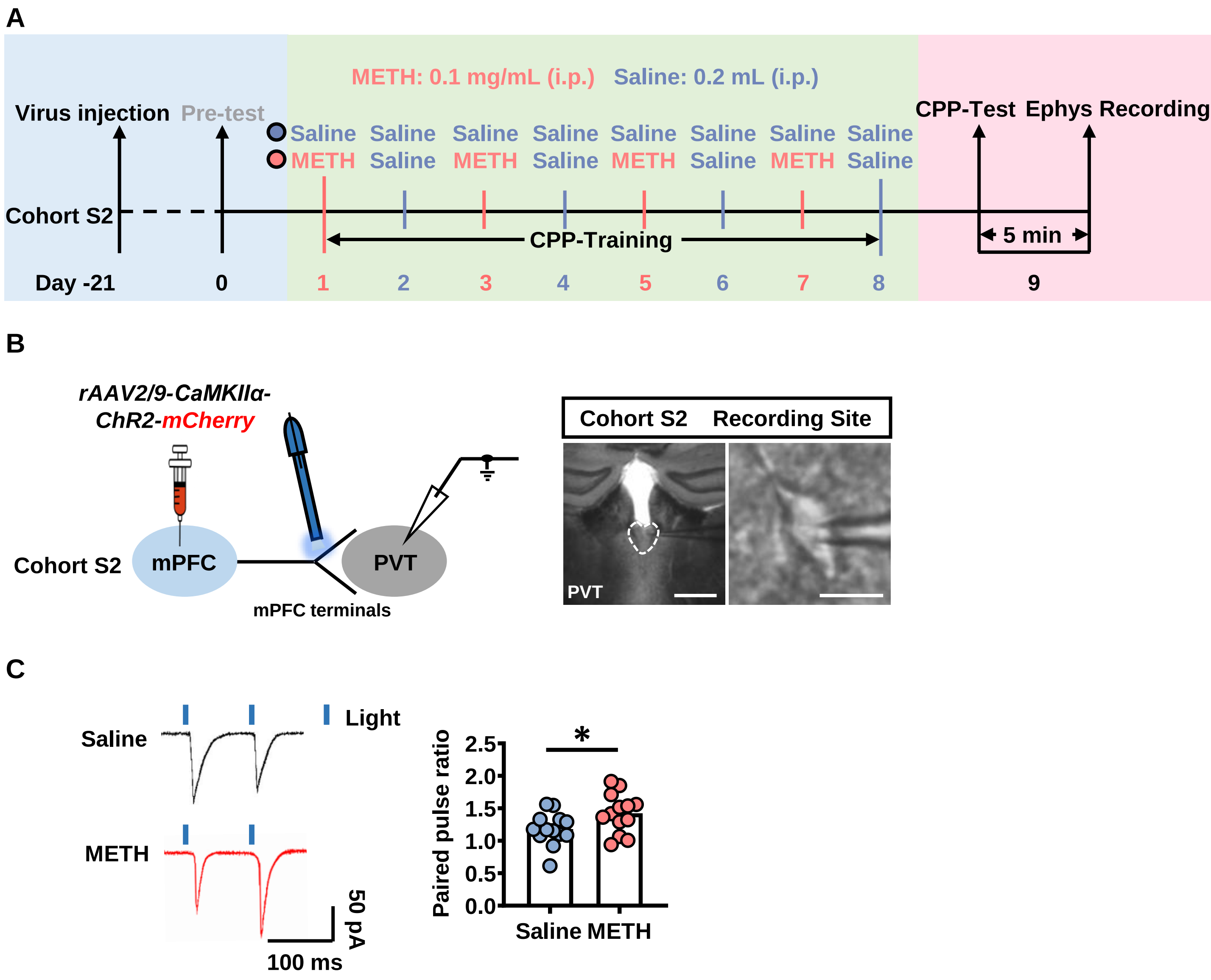

A
METH: 0.1 mg/mL (i.p.) Saline: 0.2 mL (i.p.)
Virus injection
Pre-test
Saline Saline Saline Saline Saline Saline Saline Saline
METH Saline METH Saline METH Saline METH Saline
CPP-Test
Ephys Recording
Cohort S2
CPP-Training
5 min
Day -21
0
1 2 3 4 5 6 7 8
9
B
rAAV2/9-CaMKIIα-ChR2-mCherry
Cohort S2
mPFC
PVT
mPFC terminals
Cohort S2 Recording Site
PVT
C
Saline
METH
Light
50 pA
100 ms
Paired pulse ratio
2.5
2.0
1.5
1.0
0.5
0.0
*
Saline METH

Figure S5

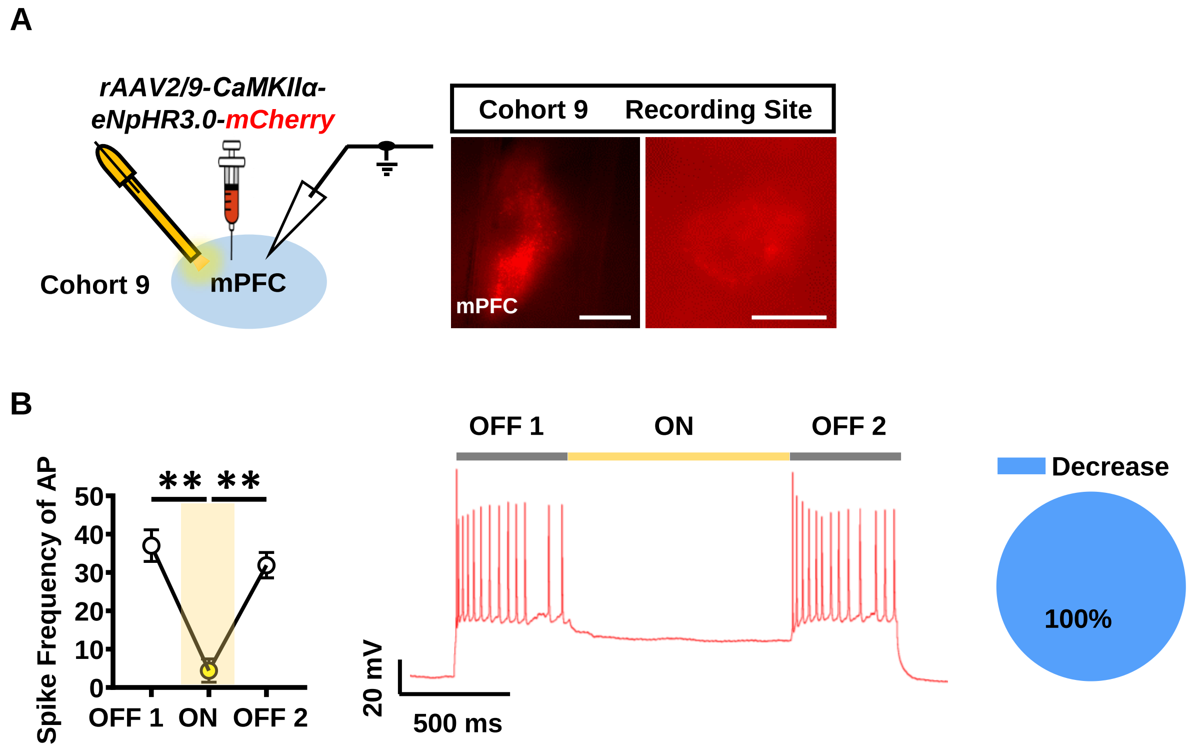
A
rAAV2/9-CaMKIIα-eNpHR3.0-mCherry
Cohort 9
mPFC
Cohort 9
Recording Site
mPFC
B
Spike Frequency of AP
50
40
30
20
10
0
OFF 1
ON
OFF 2
OFF 1
ON
OFF 2
20 mV
500 ms
Decrease
100%

Figure S6

A

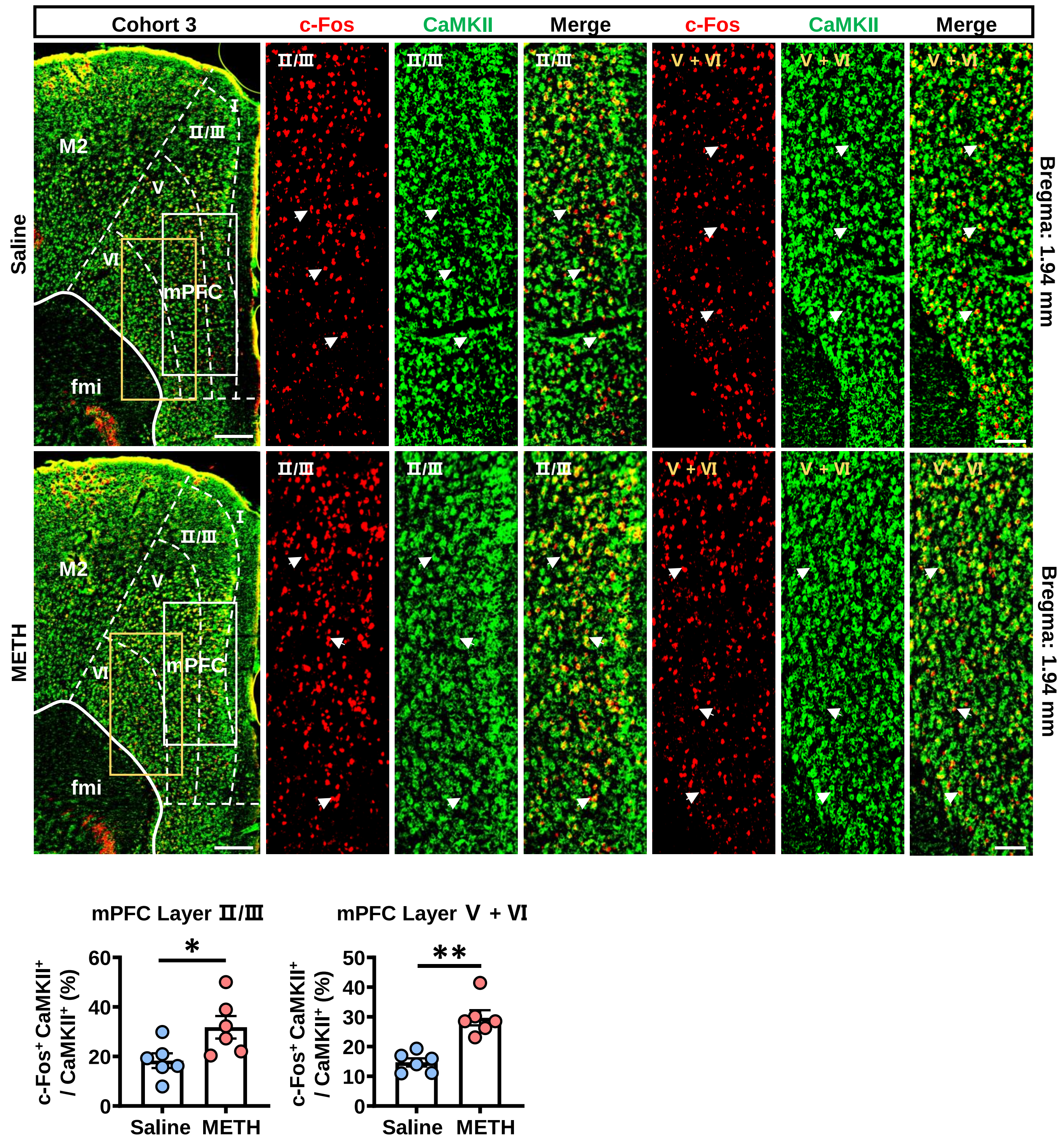

Cohort 3
c-Fos
CaMKII
Merge
c-Fos
CaMKII
Merge
Saline
METH
M2
fmi
mPFC
Bregma: 1.94 mm
Bregma: 1.94 mm
mPFC Layer Ⅱ/Ⅲ
mPFC Layer Ⅴ + Ⅵ
c-Fos⁺CaMKII⁺ / CaMKII⁺ (%)
*
**
Saline
METH

B

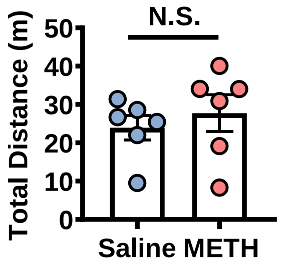

N.S.
Total Distance (m)
Saline METH

Figure S7

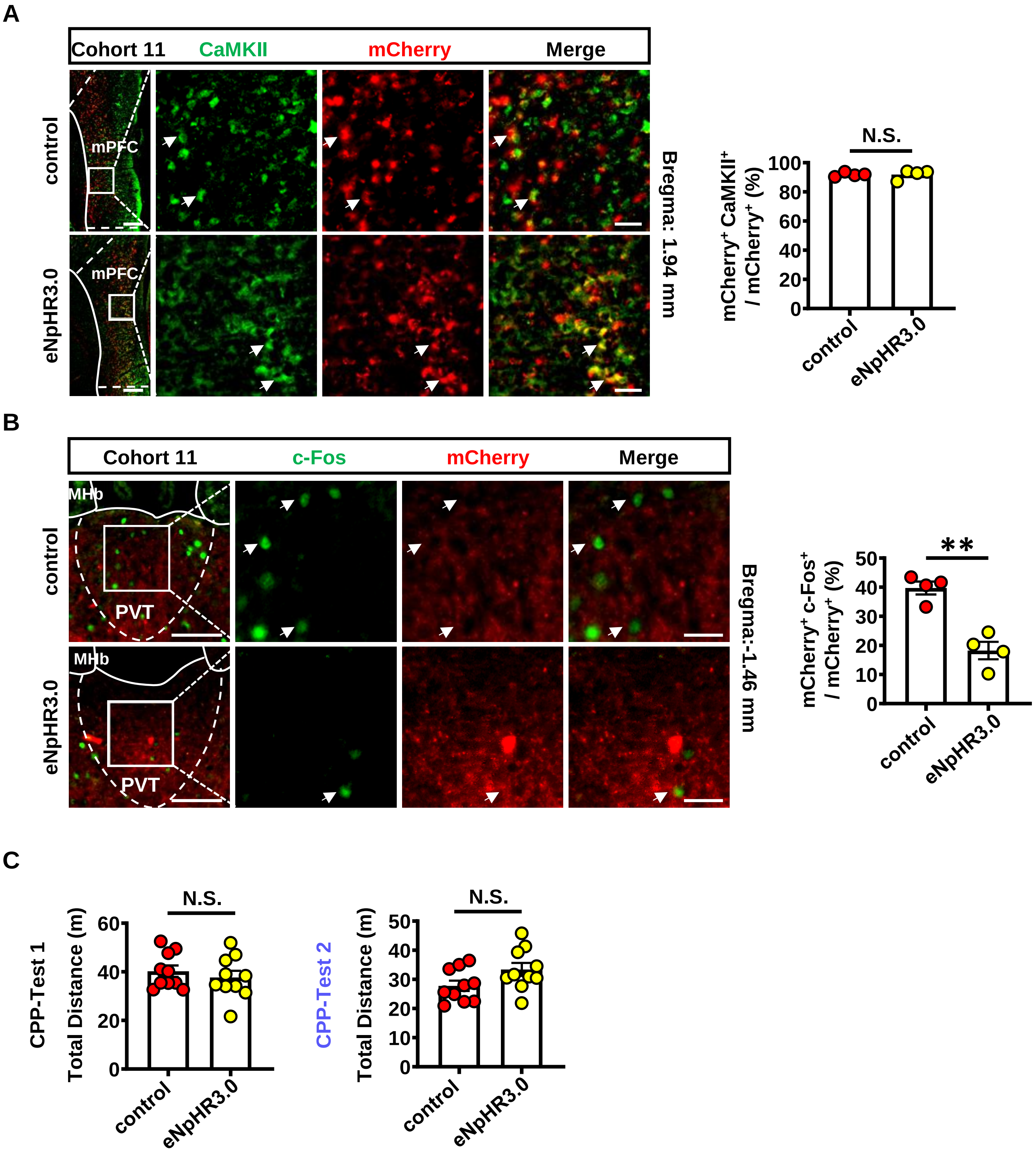
A
Cohort 11
CaMKII
mCherry
Merge
control
eNpHR3.0
mPFC
Bregma: 1.94 mm
N.S.
mCherry+ CaMKII+ / mCherry+ (%)
100
80
60
40
20
0
control
eNpHR3.0
B
Cohort 11
c-Fos
mCherry
Merge
MHb
PVT
Bregma: -1.46 mm
**
mCherry+ c-Fos+ / mCherry+ (%)
50
40
30
20
10
0
C
CPP-Test 1
Total Distance (m)
N.S.
60
40
20
0
CPP-Test 2
Total Distance (m)
N.S.
50
40
30
20
10
0

Figure S8

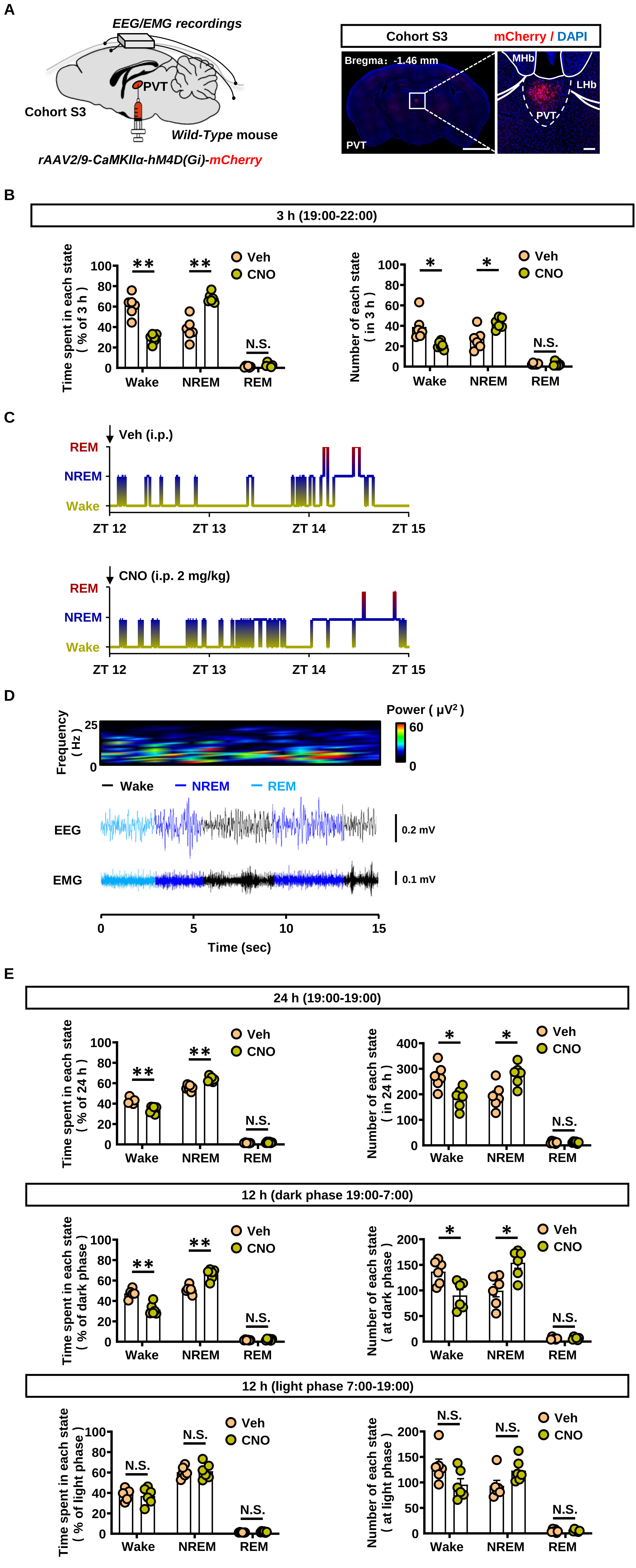
A
EEG/EMG recordings
PVT
Cohort S3
Wild-Type mouse
rAAV2/9-CaMKIIα-hM4D(Gi)-mCherry
Cohort S3 mCherry / DAPI
Bregma: -1.46 mm
PVT
MHb
LHb
PVT
B
3 h (19:00-22:00)
Time spent in each state (% of 3 h)
Number of each state (in 3 h)
Veh
CNO
Wake
NREM
REM
N.S.
C
Veh (i.p.)
CNO (i.p. 2 mg/kg)
REM
NREM
Wake
ZT 12
ZT 13
ZT 14
ZT 15
D
Frequency (Hz)
Power (μV²)
Wake
NREM
REM
EEG
EMG
0.2 mV
0.1 mV
Time (sec)
E
24 h (19:00-19:00)
Time spent in each state (% of 24 h)
Number of each state (in 24 h)
12 h (dark phase 19:00-7:00)
Time spent in each state (% of dark phase)
Number of each state (at dark phase)
12 h (light phase 7:00-19:00)
Time spent in each state (% of light phase)
Number of each state (at light phase)

Figure S9

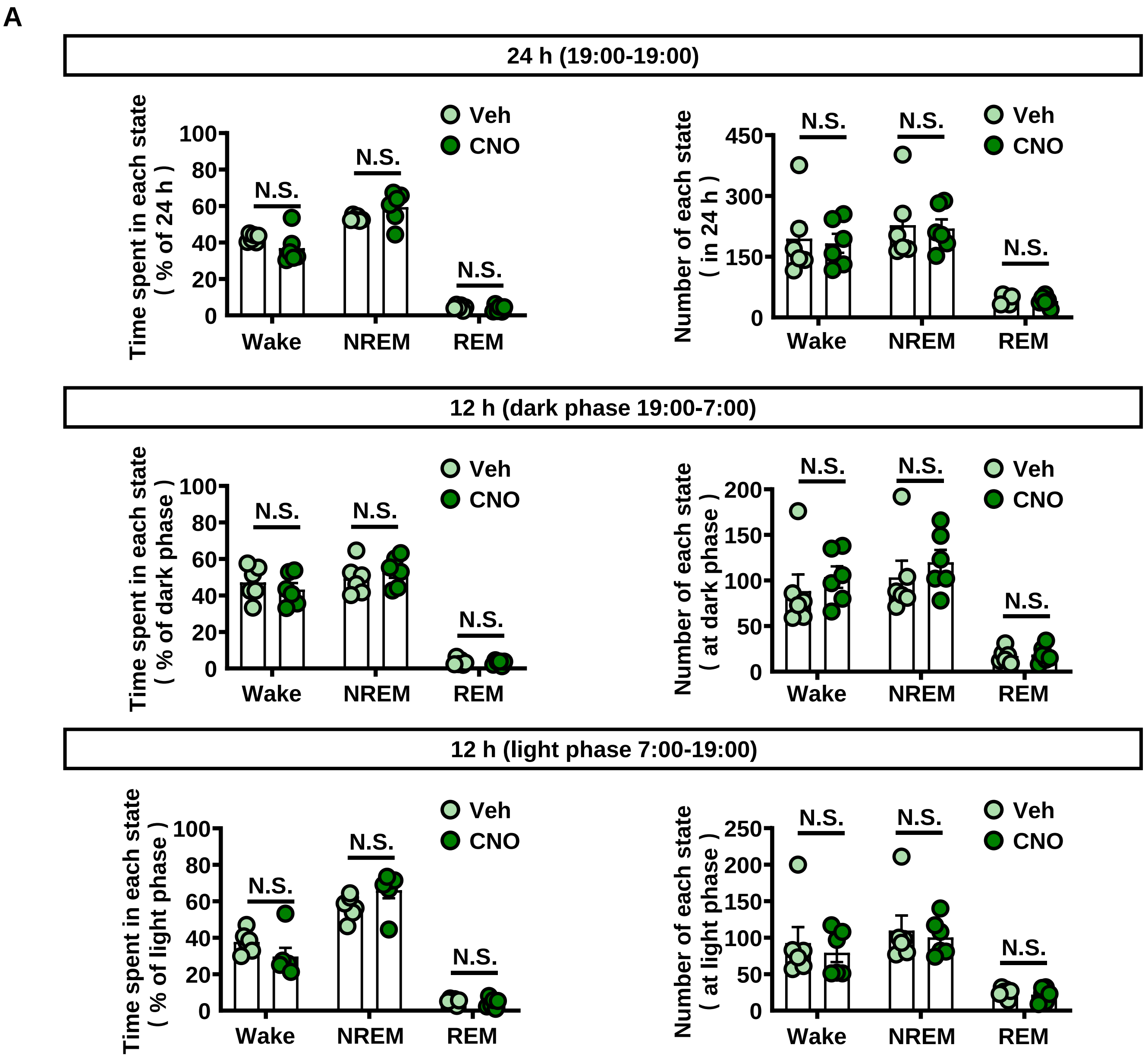
A
24 h (19:00-19:00)
Time spent in each state (% of 24 h)
Number of each state (in 24 h)
12 h (dark phase 19:00-7:00)
Time spent in each state (% of dark phase)
Number of each state (at dark phase)
12 h (light phase 7:00-19:00)
Time spent in each state (% of light phase)
Number of each state (at light phase)
Veh
CNO
N.S.
Wake
NREM
REM

Figure S10

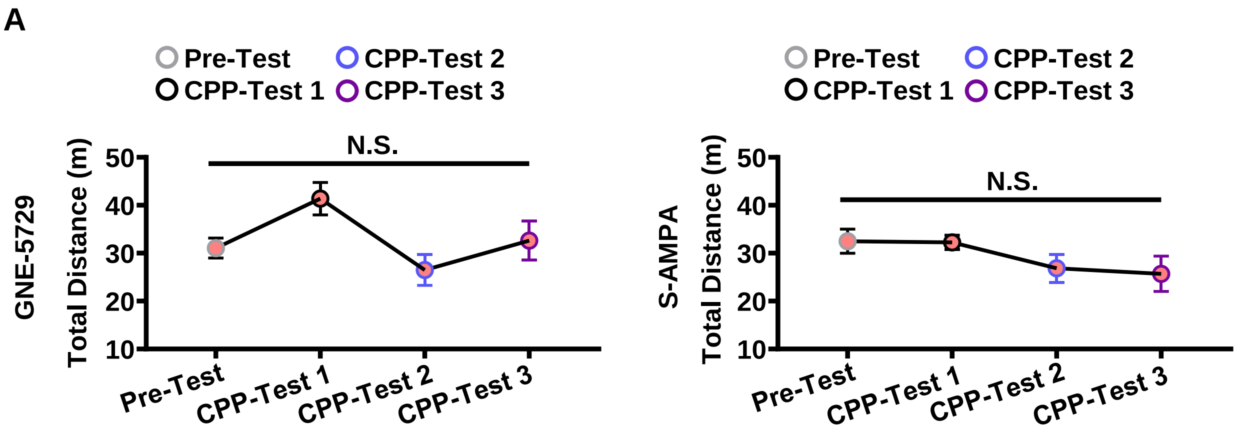

A
Pre-Test
CPP-Test 2
CPP-Test 1
CPP-Test 3
GNE-5729
Total Distance (m)
50
40
30
20
10
N.S.
Pre-Test
CPP-Test 1
CPP-Test 2
CPP-Test 3
S-AMPA
